# The biological relevance of the FspTF transcription factor, homologous of Bqt4, in *Fusarium* sp. associated with the ambrosia beetle *Xylosandrus morigerus*

**DOI:** 10.3389/fmicb.2023.1224096

**Published:** 2023-07-14

**Authors:** Nohemí Carreras-Villaseñor, Luis A. Martínez-Rodríguez, Enrique Ibarra-Laclette, Juan L. Monribot-Villanueva, Benjamín Rodríguez-Haas, José A. Guerrero-Analco, Diana Sánchez-Rangel

**Affiliations:** ^1^Laboratorios de Biología Molecular y Fitopatología, Instituto de Ecología A.C. (INECOL), Red de Estudios Moleculares Avanzados (REMAv), Xalapa, Mexico; ^2^Laboratorio de Genómica y Transcriptómica, Instituto de Ecología A.C. (INECOL), Red de Estudios Moleculares Avanzados (REMAv), Xalapa, Mexico; ^3^Laboratorio de Química de Productos Naturales, Instituto de Ecología A.C. (INECOL), Red de Estudios Moleculares Avanzados (REMAv), Xalapa, Mexico; ^4^Investigadora Por Mexico-CONAHCyT, Xalapa, Mexico

**Keywords:** transcription factor, Bqt4, *Fusarium*, *Xylosandrus morigerus*, phytopathogen

## Abstract

Transcription factors in phytopathogenic fungi are key players due to their gene expression regulation leading to fungal growth and pathogenicity. The KilA-N family encompasses transcription factors unique to fungi, and the Bqt4 subfamily is included in it and is poorly understood in filamentous fungi. In this study, we evaluated the role in growth and pathogenesis of the homologous of Bqt4, FspTF, in *Fusarium* sp. isolated from the ambrosia beetle *Xylosandrus morigerus* through the characterization of a CRISPR/Cas9 edited strain in Fsp*tf.* The phenotypic analysis revealed that TF65-6, the edited strain, modified its mycelia growth and conidia production, exhibited affectation in mycelia and culture pigmentation, and in the response to certain stress conditions. In addition, the plant infection process was compromised. Untargeted metabolomic and transcriptomic analysis, clearly showed that FspTF may regulate secondary metabolism, transmembrane transport, virulence, and diverse metabolic pathways such as lipid metabolism, and signal transduction. These data highlight for the first time the biological relevance of an orthologue of Bqt4 in *Fusarium* sp. associated with an ambrosia beetle.

## 1. Introduction

Ambrosia beetles are members of the wood-decaying ecosystem, are xylem-borers of dead and stressed trees that perform fungus farming as an ecological strategy, and carry spores of symbiotic fungi in specialized structures of their exoskeletons (the mycetangium). Once the insect is established in the plant host, the fungi colonize the drilled galleries and serve as the only source of food for both the adult beetle and the developing larvae (Six, 2012; Kirkendall et al., 2015; Huang et al., 2020; Dzurenko and Hulcr, 2022). The beetle-fungi associations are variable from the highly specific nutritional symbiosis to less specific association with other fungi acquired from the environment but which are established on the abdomen or external surfaces. Thus, fungal mycobiome is considered complex and includes phytopathogens (Bateman et al., 2016; Miller et al., 2019; Rassati et al., 2019; Hulcr et al., 2020; Morales-Rodríguez et al., 2021).

The ambrosia beetle *Xylosandrus morigerus* is an ecological generalist (Andersen et al., 2012), with expanding invasiveness (Gugliuzzo et al., 2021) damaging both agriculture and/or forestry areas. Recently, we reported the characterization of two phytopathogenic fungi isolated from *X. morigerus*, both closely related and belonging to *Fusarium solani* species complex (FSSC) but not to the Ambrosia *Fusarium* Clade (AFC), i.e., these fungi are symbiotic but maybe not strict nutritional mutualists (Bateman et al., 2016; Carreras-Villaseñor et al., 2022). Studies in *Fusarium* spp. as symbionts of ambrosia beetles are focused on its isolation and identification, phylogenetic analyzes, symbiotic interaction, and biological and chemical control (Eskalen et al., 2012; Freeman et al., 2013; O’Donnell et al., 2015; Carrillo et al., 2016, 2020; Short et al., 2017; Guevara-Avendaño et al., 2018, 2019, 2020; Kehelpannala et al., 2018; Mayorquin et al., 2018; Na et al., 2018; Grosman et al., 2019; Huang et al., 2020; Lynn et al., 2020; Takashina et al., 2020; Carreras-Villaseñor et al., 2022). However, there are scarce molecular analyzes related to genomics, transcriptomics, metabolomics, among others (Sánchez-Rangel et al., 2018; Sakai et al., 2020; Gutiérrez-Sánchez et al., 2021; Pérez-Torres et al., 2021; Ibarra-Laclette et al., 2022) that can give clues of the biological processes that are required for the infection of the plant host.

On the other hand, fungi perform transcriptional reprogramming across their life cycle (Riquelme et al., 2018; Nowrousian, 2022) and during their interaction with plants (Tan and Oliver, 2017; John et al., 2021), and it is already known that transcription factors (TFs) play a crucial role. In *Fusarium* spp., the TFs Sge1 (SIX gene expression 1), FTF1, and FTF2 are involved in the regulation of biological processes, such as hyphal differentiation to reproductive structures and the expression of effectors (Jonkers et al., 2012; van der Does et al., 2016; Rocafort et al., 2020; Zhao et al., 2020; Zuriegat et al., 2021). In other phytopathogenic fungi, TFs members of diverse families such as the Zinc (Zn) finger-binding proteins family, bZIP family, Ste12 family, Velvet family, Gti1/Pac2 family, Fork head family, and Asm1, Phd1, Sok2, Efg1, StuA (APSES) family have been characterized, finding roles in the regulation of development and pathogenesis (Tan and Oliver, 2017; John et al., 2021). Specifically, the APSES subfamily belongs to a bigger fungal TF family called the KilA-N.

The KilA-N family comprises Swi4/6, Mbp1, Res1/2, Cdc10 (SMRC), Xbp1, APSES, and Bqt4 TF subfamilies sharing the ancestral KilA-N DNA-binding domain (Medina et al., 2019). The KilA-N domain is considered homologous to the fungal DNA-binding APSES domain. It is reported to adopt the topology of a winged helix-turn-helix (wHTH) domain (Liu et al., 2015; Hu et al., 2019a,b). The KilA-N family members have diverse functions; the APSES subfamily is the most studied, and they are considered key regulators of fungal development, secondary metabolism, and virulence (Zhao et al., 2015; Medina et al., 2019). The SMRC subfamily regulates cell cycle entry and progression (Medina et al., 2016, 2019), whereas the Xbp1 subfamily is required for morphogenesis, control of cell cycle, and metabolism (Medina et al., 2019; Xin et al., 2020; Zhang et al., 2021). The Bqt4 subfamily is the youngest member of the KilA-N family; few studies are focused on this subfamily, mainly in *Schizosaccharomyces pombe* in which Bqt4 is a nuclear membrane protein (Chikashige et al., 2009) and regulates the replication of certain chromosomes regions by tethering specific heterochromatic regions ensuring the fidelity of the DNA synthesis and reassembling of the heterochromatin (Ebrahimi et al., 2018) during mitosis. In *Aspergillus nidulans*, MtgA, the homologous of Bqt4, was reported with a role in nuclear and nucleolar structure and it is required for mitotic-specific tethering to the nuclear envelope of Gle1, an mRNA export factor, but not for attachment of telomers to the nuclear envelope (Chemudupati et al., 2016). So far, the function of Bqt4 in phytopathogenic fungi has yet to be investigated at the molecular level. Therefore, we adopted *Fusarium* sp. INECOL-BM-06, which was isolated from *X. morigerus*, as a model, and we performed the characterization of an edited strain, by the CRISPR/Cas9 system, in Fsp*tf*, homologous to *Bqt4*. The phenotypic analysis of the edited strain suggests an essential role of FspTF in growth and pathogenicity. Moreover, the data obtained through high-throughput approaches suggest that FspTF regulates the expression of genes involved in key biological processes such as secondary metabolism, membrane transport, regulation of gene expression, and lipid metabolism, among others.

## 2. Materials and methods

### 2.1. Fungal strains, media, and growth conditions

*Fusarium* sp. INECOL_BM-06 (Carreras-Villaseñor et al., 2022) was used as the wild-type (WT) strain throughout this work. *Fusarium* sp. WT and mutant strains were propagated on potato dextrose agar (PDA, DIFCO) at 25°C. For stress responses assays, a minimal medium (MM) was prepared as described by Leslie and Summerell (2006) and supplemented with H_2_O_2_ 0.08%, SDS 0.025%, Congo Red (CR) 0.02%, Sorbitol 1 M, KCl 0.7 M, or hydroxyurea (HU) 20 mM. The MM was supplemented with bacteriological Agar (SIGMA) 2%. A plug (5 mm in diameter) from the edge of the actively growing colony of the strains grown in PDA was inoculated in the center of the plate. The cultures were incubated at 25°C for 7 days. The experiment was performed with three technical replicates.

### 2.2. Sequences analyzes

Protein structure analyzes and domain identifications were performed in SMART^1^ (Letunic and Bork, 2018), NCBI CDD (Lu et al., 2020)^2^ and InterPro^3^ (Paysan-Lafosse et al., 2023) with default parameters to search for conserved domains. Gene structure, mRNA, and protein sequence prediction of edited Fsp*tf* was done with the FGENESH program^4^ (Solovyev et al., 2006).

The amino acid sequences of KilA-N proteins from *Fusarium* sp. INECOL_BM-06 were retrieved from the unpublished assembled genome provided by Ibarra-Laclette and Sánchez-Rangel from the Institute of Ecology (INECOL) at Xalapa, Veracruz-Mexico using Swi4, Swi6, StuA, and MgtA from *Aspergillus nidulans* as the query in BLASTP searches. Homologous KilA-N domain-containing proteins from *Schizosaccharomyces pombe*, *Aspergillus nidulans*, *Neurospora crassa*, *Magnaporthe oryzae*, *Saccharomyces cerevisiae*, *Ustilago maydis*, *Cryptococcus neoformans*, *Fusarium graminearum*, *Fusarium kuroshium*, and *Fusarium euwallacea* were obtained by BLASTP search of non-redundant protein sequence (nr) database in the National Center of Biotechnology Information (NCBI)^5^; *Fusarium solani* homologous sequences were obtained from Mycocosm Database^6^ (Supplementary Table S1). Multiple sequence alignments and phylogenetic trees were performed in Seaview (Gouy et al., 2010) using the neighbor-joining (NJ) method, while tree reliability was estimated by bootstrap analysis with a total of 1,000 pseudoreplicates. For the identification of secreted hydrolytic enzymes, amino acids sequences were analyzed with SignalP 6.0 (Teufel et al., 2022)^7^ and DeepLoc 2.0 (Thumuluri et al., 2022).^8^ The prediction of apoplastic or cytoplasmatic effectors was performed with EffectorP 3.0^9^ (Sperschneider and Dodds, 2022). The classification in protein domain families was achieved by searches in the Conserved Domain Database (CDD) (Lu et al., 2020).^10^

### 2.3. Expression analysis by quantitative RT-PCR in fungal and plant tissue

Total RNA from fungal and plant tissue was isolated by phenol extraction and LiAc precipitation. Primers for RT-qPCR were designed to produce amplicons around 150 bp using the IDT PrimerQuest™ tool (Supplementary Table S2). cDNA was synthesized using SuperScript III Reverse Transcriptase (Invitrogen) following the manufacturer’s instructions. The quantitative PCR was carried out according to the SYBR Green PCR Master Mix protocol (Applied Biosystems) using the Mx3000P qPCR system (Agilent). *EF1-a* was used as the standard (housekeeping gene) to normalize the content of cDNA, and the 2^-ΔΔCt^ method (Livak and Schmittgen, 2001) was employed to determine the relative expression and compare the treated samples.

### 2.4. Protospacer selection, CRISPR/Cas9 plasmid construction, genome edition confirmation, and deletion construct generation

The exons of Fsp*tf* were scanned by CRISPOR^11^ (Concordet and Haeussler, 2018) for the prediction of accurate guides for gene edition by the CRISPR/Cas9 system. Guide 65Fw (ACACCTAAGATGGTCGGCAC) for exon 2 was selected based on its on-target scores and no off-target prediction. The 65Fw protospacer sequence was inserted in plasmid pFC332 by combining two PCR fragments amplified from plasmid pFC334 and the linearized pFC332 plasmid in a NEBuilder reaction (Nødvig et al., 2015; Wenderoth et al., 2017). The primer sequences are listed in Supplementary Table S2. The fragments were amplified from pFC334 with Platinum Taq DNA Polymerase High Fidelity (Invitrogen) following the instruction of the manufacturer with a specific PCR program (First denaturation 94°C for 2 min, 30 cycles of 94°C for 15 s, 63°C for 30 s, 68°C for 30 s, and final elongation 68°C for 10 min). The plasmid pFC332 was linearized with PacI (Invitrogen) and assembled with the purified PCR fragments, following the NEBuilder protocol. DNA sequencing corroborated the plasmid construct. The edited strain was isolated, and the genome edition was confirmed by the amplification of a 1,524 bp fragment with primers that anneal specific sites up- and downstream of the protospacer sequence with posterior sequence analysis (Supplementary Table S2). The deletion construct was generated employing double-joint PCR (Yu et al., 2004) containing the hygromycin phosphotransferase gene (*hph*) as a selection marker, which confers hygromycin resistance, joined with the 5′ and 3′ flanking regions of the *Fusarium* sp. *tf* gene. The *hph* selection marker was amplified from pCB1004 and the 5′ and 3′ flanking regions from the genomic DNA of *Fusarium* sp. INECOL_BM-06 strain. The final construct was introduced in *Fusarium* sp. INECOL_BM-06 strain. The replacement mutant was isolated and confirmed with primers annealing to the ORF by PCR. The primers used are listed in Supplementary Table S2.

### 2.5. Transformation of *Fusarium* sp. protoplast

*Fusarium* sp. was transformed as described previously (Connolly et al., 2013), with some modifications. Briefly, fungal conidia were harvested from a carboxymethylcellulose (CMC) culture (Cappellini and Peterson, 1965) and inoculated in 50 mL of YPD broth for overnight cultivation at 28°C and 200 rpm. The mycelium was harvested by filtering, washed with KCl solution (1.4 M), and digested in a lysing enzyme (Sigma) suspension (40 mg/mL in KCl solution 1.4 M) for 3 h with soft shaking at 70 rpm at 28° C. Protoplast were separated from cell fragments by filtering through to 30 μm membrane and sedimented by centrifugation at 8000 rpm for 10 min at 4°C. The lysing enzyme solution was discarded, and the protoplast was washed once with STC (Sorbitol 1.2 M, Tris–HCl pH 8 10 mM, and CaCl_2_ 50 mM) and resuspended in 400 μL of STC. Then, 10 μg of DNA and 100 μL of PEG 30% were added to the protoplast followed by 20 min incubation at room temperature. We then added 1 mL of PEG 30%, followed by 5 min incubation at room temperature. Finally, 2 mL of STC was added. Half of the suspension was mixed with warm 30 mL regeneration medium and split into two Petri dishes. The operation was repeated with the rest of the suspension. After overnight incubation at 28°C, the transformation plates were overlayed with a 15 mL warm regeneration medium containing hygromycin (100 μg/mL). Resistant colonies were picked and purified to obtain homokaryotic strains.

### 2.6. Measurement of germination rate and yield of conidia

For conidia generation, three mycelium plugs were inoculated in 25 mL of CMC and incubated at 28°C and 200 rpm for 7 days. Then, they were harvested and counted using a hemocytometer. To examine conidia germination levels, conidia of WT and mutants were inoculated into 5 mL of MM at a concentration of 2×10^5^ conidia/mL and incubated at 28°C. Then, 2 h post-inoculation (hpi), germination was assessed every 2 h. The percentage of germination was calculated by the number of total conidia and germinated conidia visualized in the hemocytometer.

### 2.7. Pathogenesis assays

Foliar disk of 2 cm in diameter from freshly collected leaves from *Populus nigra*, *Coffea arabica* cv. Marsellesa, *Citrus sinensis*, and *Citrus latifolia* were injured in the center and inoculated with a mycelium plug from the edge of an actively growing colony of the strains grown in PDA. The foliar disks were placed in wet chambers and incubated at 25°C ± 1 with photoperiod (16 h dark/8 h light) for 7 days. Twelve disks were inoculated with each strain. Symptom development was recorded every day. The lesion area was measured by Image J conducting particle quantification based on image contrast.

### 2.8. Plant cell death and reactive oxygen species detection

Plant cell death was evaluated by staining with trypan blue (Fernández-Bautista et al., 2016). ROS, specifically H_2_O_2_, was determined by DAB staining (Daudi and O’Brien, 2012) after 4 days of infection. Images were acquired with a Zeiss SteREO Discovery.V8 stereomicroscope.

### 2.9. Untargeted metabolomics assays

The WT and TF65-6 strains were grown in PDA for 7 days in the dark after the mycelium was collected and frozen immediately. For sample preparation, 0.3 g of dried mycelial samples and 30 mL of methanol (HPLC grade, Sigma-Aldrich) were used. Methanolic extracts were obtained by four pulses of 10 min each in an ultrasonic bath (Cole Parmer, United States). The supernatant was recovered and concentrated to dryness in a rotatory evaporator (RII, Büchi, Switzerland). Dried methanolic extracts were redissolved in methanol (50 mg/mL, MS grade with 0.1% of formic acid, Sigma-Aldrich), filtered with 0.2 μm PTFE membranes, and placed in 2 mL Ultra Performance Liquid Chromatography (UPLC) vials. The untargeted metabolomics analyzes were performed in a UPLC (Class I, Waters™, United States) coupled to a quadrupole-time-of-flight Synapt G2-Si mass spectrometer (Waters, United States). The chromatography was carried out on a Waters Acquity BEH column (1.7 μm, 2.1 × 50 mm) with column and sample temperatures of 40 and 15°C, respectively. The mobile phase consisted of (A) water and (B) acetonitrile, both with 0.1% of formic acid (Sigma-Aldrich, United States). The gradient conditions of the mobile phases were 0–20 min, a linear gradient of 1–99% B, 20–24 min 99% B isocratic, and a 24–25 min linear gradient of 90–1% B (total run time 30 min). The flow rate was 0.3 mL/min, and 5 μL of the extract was injected. The mass spectrometric analysis was performed with an electrospray ionization source in positive and negative mode with a capillary, sampling cone, and source offset voltages of 3,000, 40, and 80 V, respectively. The source temperature was 120°C, and the desolvation temperature was 20°C. The desolvation gas flow was 600 L/h, and the nebulizer pressure was 6.5 Bar. Leucine-enkephalin was used as the lock mass [556.2771, (M + H)^+^; 554.2615, (M-H)^−^]. The conditions used for MSe analysis were: mass range 50–1,200 Da, Function 1 CE, 6 V, function 2 CER 10–30 V, and scan time of 0.5 s. The data were acquired and processed with MassLynx (version 4.1) and MarkerLynx (version 4.1) softwares from Waters. The tentative identification of metabolites was performed using the MetaboAnalyst bioinformatics platform.^12^ The Statistical Analysis module was used for fold change (FC) analyzes to identify over- and down-accumulated metabolites in the mutant strain (log2FC = ±1).

### 2.10. RNA-seq and differential gene expression analysis

The mycelium of the WT and TF65-6 strains analyzed in the transcriptomic experiment was collected and frozen immediately after growing in PDA for 7 days in the dark. Total RNA was extracted using the TRIzol protocol (Invitrogen). Following the manufacturer’s instructions, six libraries (three biological replicates for each strain) were prepared with the TruSeq RNA Sample Preparation Kit (Illumina, San Diego, CA, United States). The six libraries were sequenced using the NextSeq500 platform (Illumina, San Diego, CA, United States) on paired-end reads at 2 × 150 bp format. The raw data obtained from RNA-seq were deposited in the Short Read Archive (SRA) database of the National Center for Biotechnology Information (NCBI), accession PRJNA972669.

Raw paired-end reads were analyzed with Trimmomatic v0.38 (Bolger et al., 2014) to filter out low-quality sequences. Reads mapping to the reference genome (*Fusarium* sp. isolate INECOL_BM-06, unpublished assembled genome, access provided by Ibarra-Laclette and Sánchez-Rangel from the INECOL at Xalapa, Veracruz-Mexico) and transcript abundance estimation were performed using Bowtie2 v2.3.5.1 mapper (Langmead and Salzberg, 2012) and Expectation–Maximization (RSEM) v1.3.1 (Li and Dewey, 2011) software, respectively. A transcript abundance matrix was created containing each of *Fusarium* sp. INECOL_BM-06 genes (rows) and the expected counts (EC). In addition, transcripts per million (TPM) values were calculated for both WT and TF65-6 strains (columns). EC values represent the expression levels calculated by the maximum likelihood estimation approach and posterior mean estimates with 95% credibility intervals. For its part, TPM values can be considered highly consistent when a comparison is performed among different samples (Wagner et al., 2012). These values were used to perform principal component analysis (PCA) and a negative binomial model was used to identify differentially expressed genes (DEGs) that were used to perform pairwise Wald tests. In addition, the Benjamini–Hochberg multiple testing was performed with DESeq2 v1.2.4.0 R/Bioconductor package (Love et al., 2014). A log2 Fold Change (FC) value ±1.0 and an adjusted value of *p* of ≤0.05 were used as threshold criteria.

### 2.11. Annotation of the differentially expressed genes and gene ontology enrichment

The annotation of the DEGs was performed by homology-based inference. The annotation process involved BLASTp similarity searches (e-value cutoff of 10^−5^) against the proteins reported for *Fusarium solani*, *Fusarium oxysporum* NRRL 3293, *Fusarium fujikuroi* IMI 58289, *Fusarium verticillioides* 7600, and *Fusarium graminearum* PH-1. *Neurospora crassa* and *Saccharomyces cerevisiae* were also added. All protein sets were downloaded from the latest version available on the GenBank database.^13^ GO terms were assigned by transferring GO terms from the *Fusarium* species mentioned above using the software package InterProScan (Jones et al., 2014).

The functional enrichment analysis was performed considering the list of differentially expressed genes using the web tool g:Profiler (Raudvere et al., 2019).^14^ The g:SCS method was applied to perform multiple testing corrections using the p-adjusted values with a ≤ 0.05 threshold. The available *Fusarium* species on web site (*Fusarium graminearum* str. PH-1, *Fusarium fujikuroi*, *Fusarium oxysporum* f. sp. *lycopersici* 4,287, *Fusarium pseudograminearum* CS3096, *Fusarium vanettenii* 77–13-4 (*F. solani*), and *Fusarium verticillioides* 7600) were used as references.

### 2.12. Statistical analyzes

All the experimental data collected were statistically evaluated using the GraphPad Prism 9.5.1 software (GraphPad Software Inc., San Diego, United States), with *p* < 0.05 considered statistically significant in all the cases.

## 3. Results

### 3.1. Identification of members of the KilA-N/APSES transcription factor family in *Fusarium* sp. associated with *Xylosandrus morigerus*

**Figure 1:**
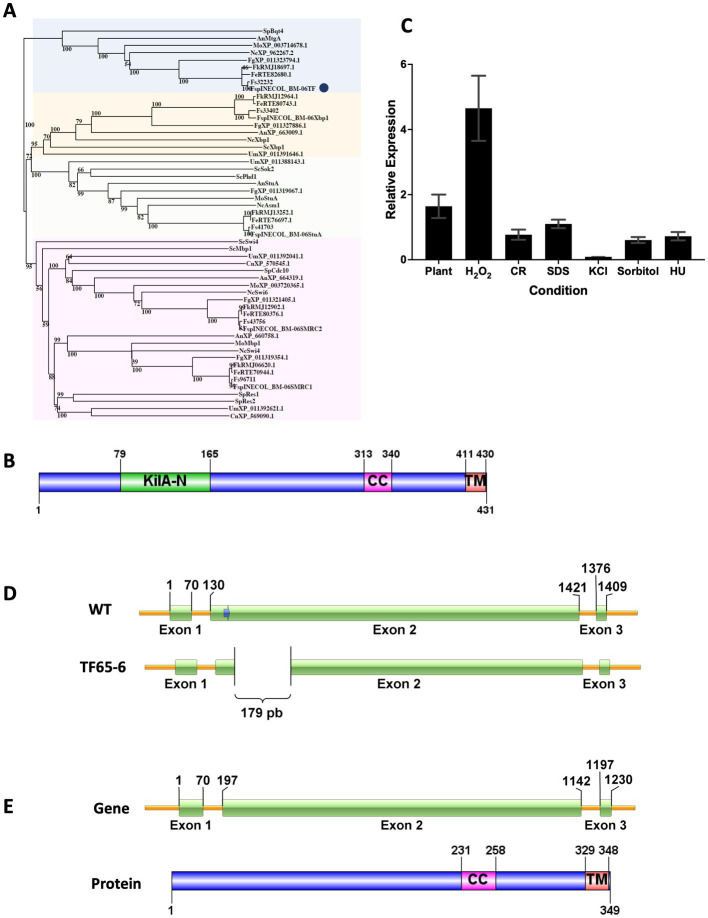
Protein sequence analyzes of KilA-N/APSES TF, Expression of Fsp*tf* of *Fusarium* sp. INECOL_BM-06 and Gene Deletion Event in TF65-6 mutant obtained with the CRISPR/Cas9 system. **(A)** Phylogenetic tree based on KilA-N proteins of different fungi. Numbers in nodes indicate the bootstrap value (expressed as percentages of 1,000 replications). The cluster highlighted in blue encloses Bqt4 homologs. TF of *Fusarium* sp. is marked by a blue circle. The cluster highlighted in yellow encloses Xbp1 homologs. The cluster highlighted in orange encloses StuA homologs. The cluster highlighted in pink encloses Swi4/Swi6 homologs. Sp: *Schizosaccharomyces pombe*, An: *Aspergillus nidulans*, Fg: *Fusarium graminearum*, Nc: *Neurospora crassa*, Mo: *Magnaporthe oryzae*, Fs: *Fusarium solani*, Sc: *Saccharomyces cerevisiae*, Um: *Ustilago maydis*, Cn: *Cryptococcus neoformans*, Fe: *Fusarium euwallacea*, Fk: *Fusarium kuroshio*, Fsp: *Fusarium* sp. INECOL_BM-06 **(B)** Domain organization of TF of *Fusarium* sp. INECOL_BM-06. KilA-N: DNA-binding domain. CC: coiled-coil region. TM: transmembrane helix region. **(C)** Relative expression of Fsp*tf* in different culture conditions. Expression is relative to *EIF1-a* expression and control condition. Bars are average ± SD of three technical replicates. Basal expression = 1. **(D)** The Fsp*tf* gene structure and the location of the protospacer+PAM (blue arrow) sequence in exon 2. Sequence analysis of the transformant showed deletion of 179 bp in exon 2 of TF65-6. The scheme shows the deleted region. **(E)** Predicted gene structure and protein domain organization in TF65-6 strain. Notice the smaller size of exon 2 in the gene and the absence of the KilA-N domain in the protein.

To identify the KilA-N/APSES family in *Fusarium* sp. INECOL_BM-06, amino acid sequences of the characterized KilA-N proteins from *Aspergillus nidulans* were used as queries in BLASTP searches in *Fusarium* sp. INECOL_BM-06 database (unpublished data). Five proteins containing the KilA-N domain were identified in *Fusarium* sp. (Supplementary Table S3). The phylogenetic tree grouped the fungal sequences into four clades (Figure 1A). The bigger clade comprised members of the subfamily SMRC such as Swi4 and Mbp1 of *S. cerevisiae*, and Res1 and Res2 of *S. pombe*. In this clade, two sequences of *Fusarium* sp. were identified, named SMRC1 and SMRC2, which are closer to Swi4 and Swi6 of *N. crassa*, respectively. Another clade grouped the proteins of the APSES subfamily members, homologous to StuA of *A. nidulans*; this clade included one sequence from *Fusarium* sp. denominated as StuA. A third clade enclosed the sequences homologous to Xbp1 of *S. cerevisiae*, in which a sequence of *Fusarium* sp. named Xbp1 was included. The last clade grouped the proteins homologous to Bqt4 of *S. pomb*e and MtgA of *A. nidulans*, which have been previously characterized (Chikashige et al., 2009; Chemudupati et al., 2016). The *Fusarium* sp. sequence, denominated as FspTF, was more related to the *N. crassa*, *F. graminearum*, and *M. oryzae* proteins whose function has not yet been determined. Since the family Bqt4 is the youngest and least distributed and, therefore, least studied member of the family KilA-N/APSES in filamentous fungi, we characterized this gene in *Fusarium* sp. associated with *X. morigerus*. FspTF is a protein with 431 aa, Mw of 47.5 kDa, and pI 5.4. The analysis by SMART (Letunic and Bork, 2018) identified a KilA-N domain (IPR018004) in the N-terminal region (residues 79–165), a coiled-coil region (residues 313–340), and a single-pass C-terminal transmembrane helix region (residues 411–430) (Figure 1B).

### 3.2. Fsp*tf* is responsive to different stress conditions

To infer the biological function of FspTF, the expression of Fsp*tf* was evaluated during different growth conditions. Fsp*tf* expression was induced during oxidative stress, and plant infection process with 4.7 and 1.6-fold, respectively. Conversely, it was repressed in 30% in response to cell wall (CR 0.02%) stress, and in osmotic stress exerted by KCl 0.7 M, sorbitol 1 M, and DNA stress (HU 20 mM), the repression was 91, 39, and 28%, respectively. Fsp*tf* was not responsive to the growth in SDS 0.025% (Figure 1C). These data suggest a role of Fsp*tf* during stress responses and pathogenesis.

### 3.3. Fsp*tf* gene edition by the CRISPR/Cas9 system in *Fusarium* sp. results in a DNA region deletion

Once the homokaryotic strain TF65-6 was obtained after the protoplast transformation of *Fusarium* sp., sequence analysis of its genomic DNA revealed a genomic deletion of 179 bp in exon 2 (Figure 1D; Supplementary Figure S1, S2). Fsp*tf* gene structure and protein domain organization in the edited strain TF65-6 were predicted using FGENESH (Solovyev et al., 2006) and SMART (Letunic and Bork, 2018), respectively. In TF65-6, the deletion of 179 bp in exon 2 produced a frameshift that generated a longer intron 1 and smaller exon 2. This edited gene was predicted to encode a protein with 349 amino acids lacking the KilA-N DNA-binding domain but keeping the coiled-coil and transmembrane helix regions (Figure 1E). The expression analysis in growth in PDA, MM, oxidative stress, and in interaction with plant tissue indicated that Fsp*tf* was downregulated by 74, 60, 70, and 67%, respectively, in TF65-6 in comparison with the expression in the WT (Supplementary Figure S3).

### 3.4. Phenotypic characterization of Fsp*tf* edited strain

#### 3.4.1. FspTF controls development in *Fusarium* sp.

**Figure 2:**
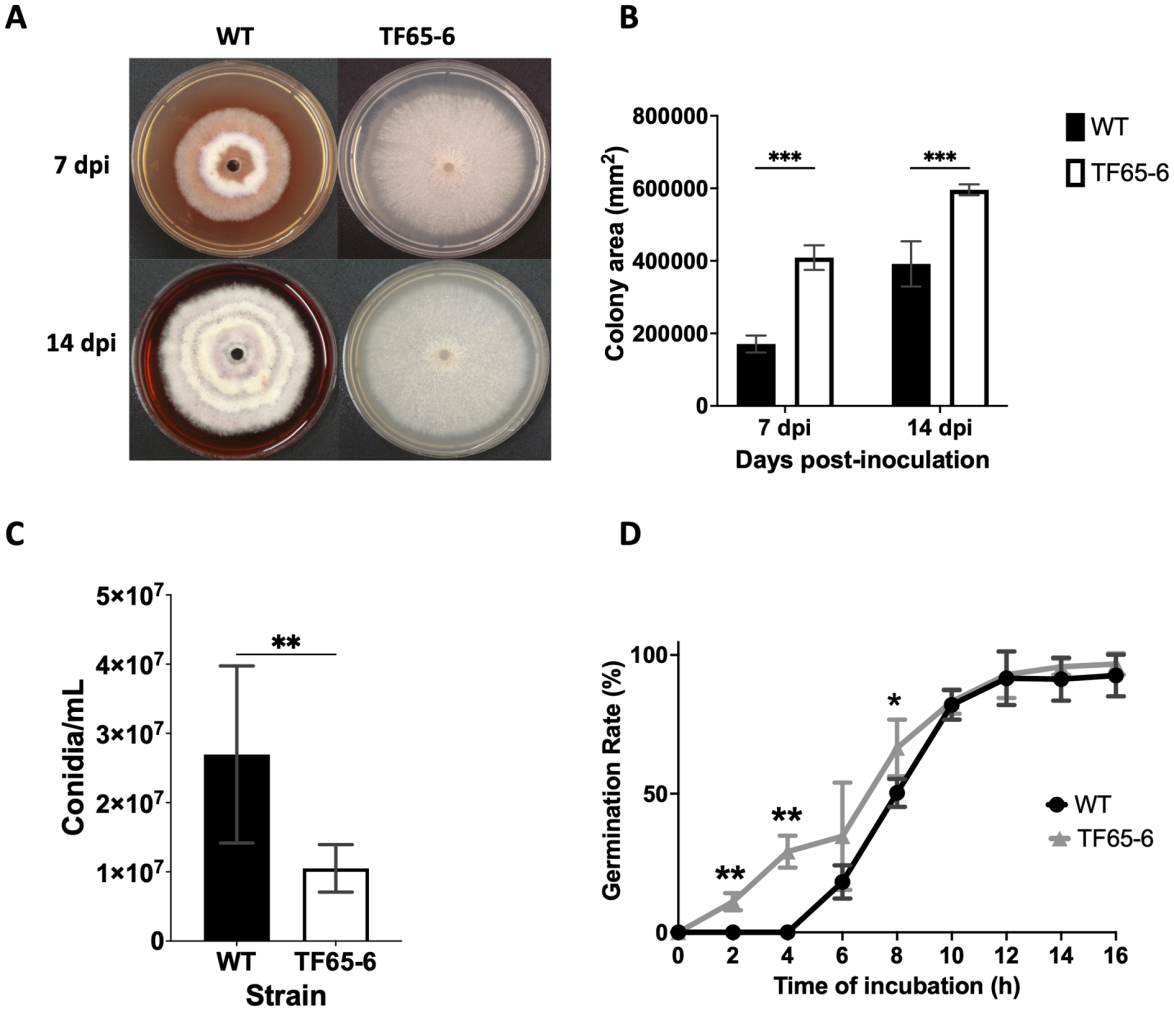
FspTF regulates development in *Fusarium* sp. INECOL_BM-06. **(A)** Growth of *Fusarium* sp. INECOL_BM-06 WT and TF65-6 in PDA. **(B)** Colony area of *Fusarium* sp. INECOL_BM-06 WT and TF65-6 in PDA. Data were obtained at 7 and 14 dpi. Bars are the average ± SD of two biological replicates with four technical replicates each. **(C)** Conidia quantification in *Fusarium* sp. INECOL_BM-06 WT and TF65-6. Conidia were obtained after 7 days of incubation in liquid CMC. Bars are average ± SD of three biological replicates with three technical replicates each (**value of *p*<0.001 by *t*-test). **(D)** Germination rates of *Fusarium* sp. INECOL_BM-06 WT and TF65-6 when inoculated in liquid MM at 28°C. The number of conidia showing germ-tube protrusion was recorded at 2 h intervals and is represented as a percentage of the total number of conidia counted with the hemocytometer. Data are presented as the average ± SD of three independent experiments with two technical replicates each. (***value of *p*<0.001, **value of *p*<0.01, *value of *p*<0.05 by Two-way ANOVA with Bonferroni multiple comparison correction).

**Figure 3:**
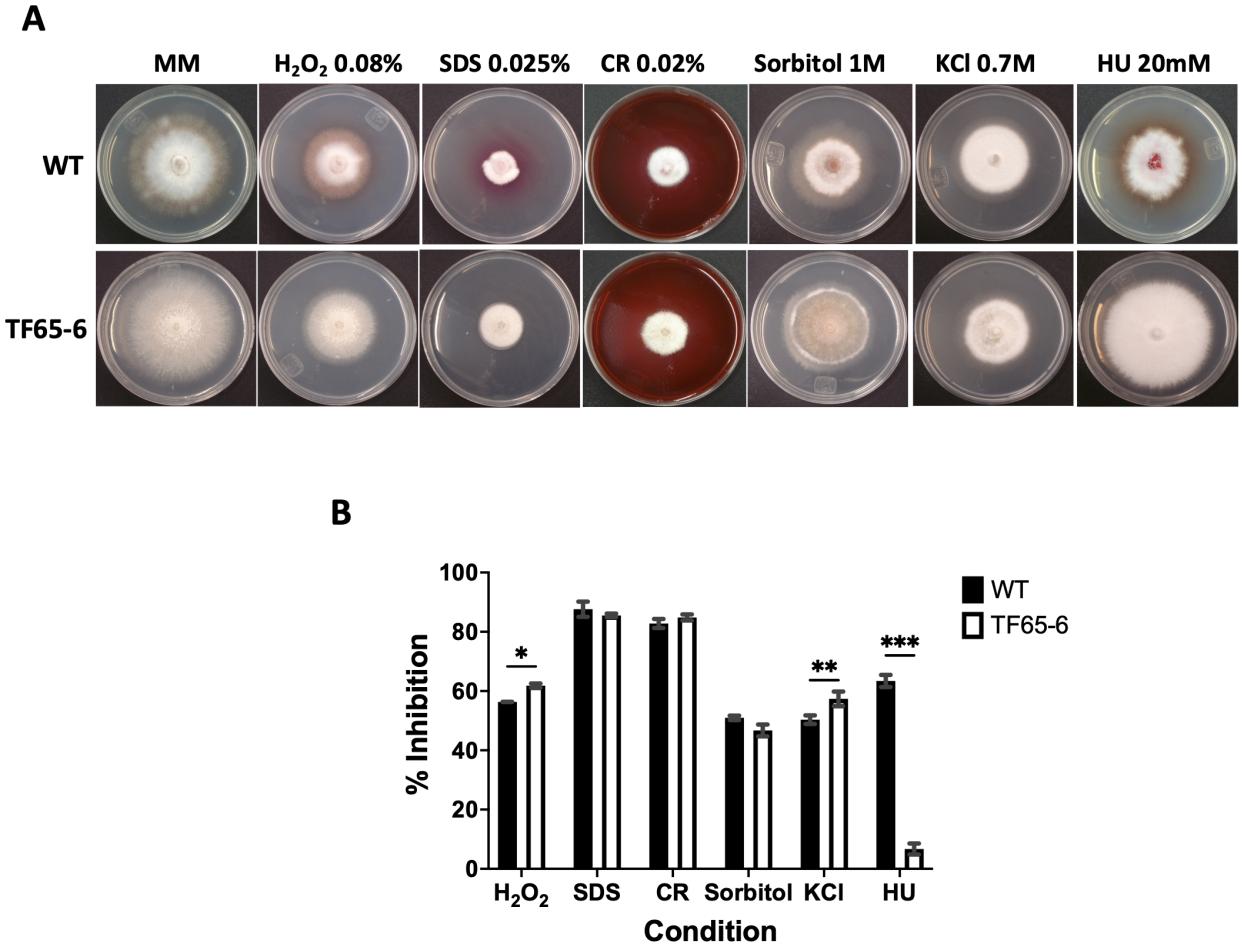
Response to different stress conditions of *Fusarium* sp. WT and TF65-6. **(A)** Growth of *Fusarium* sp. INECOL_BM-06 WT and TF65-6 in MM amended with H_2_O_2_ 0.08%, Sorbitol 1 M, SDS 0.025%, CR 0.02%, KCl 0.7 M, or HU 20 mM. **(B)** Mycelial growth inhibition of INECOL_BM-06 WT and TF65-6 grown in MM amended with H_2_O_2_ 0.08%, Sorbitol 1 M, SDS 0.025%, CR 0.02%, KCl 0.7 M, or HU 20 mM. Data were obtained at 7 dpi. Bars are average ± SD of three technical replicates. (***value of *p*<0.001; **value of *p*<0.01; *value of *p*<0.05 by Two-way ANOVA with Bonferroni multiple comparison correction).

The colony morphology of the edited strain, TF65-6, was contrasting to the WT. The WT strain presented pigmented mycelium with a concentric ring of aerial mycelium and secreted a compound that pigmented the culture medium; in contrast, TF65-6 developed white mycelium without aerial mycelium and lost pigmentation in both mycelium and medium. At 14 dpi, the contrasting phenotypes between both strains were maintained (Figure 2A). Additionally, the colony area of the TF65-6 strain was 2.4 and 1.5-fold bigger than that of the WT at 7 and 14 dpi, respectively (Figure 2B). Conidia production was affected in TF65-6, with a 58% lower quantity (Figure 2C); however, the germination rate increased, specifically, in the early stages, with 11 and 30% higher rates at 2 and 4 hpi, respectively (Figure 2D). To investigate whether FspTF has a role in stress responses, *Fusarium* sp. WT and edited strain were grown in MM amended with compounds known to cause different types of stress, such as oxidative and osmotic, and cell wall, membrane, and DNA damage (Figures 3A,B). In the presence of H_2_O_2_ (0.08%), the growth of the WT was reduced by 56%, and in TF65-6 it was reduced by 62%. In osmotic stress induced by KCl 0.7 M, TF65-6 was more sensitive with 57% of inhibition, and WT had 50% of inhibition. Interestingly, TF65-6 was more tolerant to DNA damage triggered by HU 20 mM, with 7% inhibition compared with 63% of inhibition in the WT (Figure 3B).

#### 3.4.2. Pathogenesis of *Fusarium* sp. is regulated by FspTF

**Figure 4:**
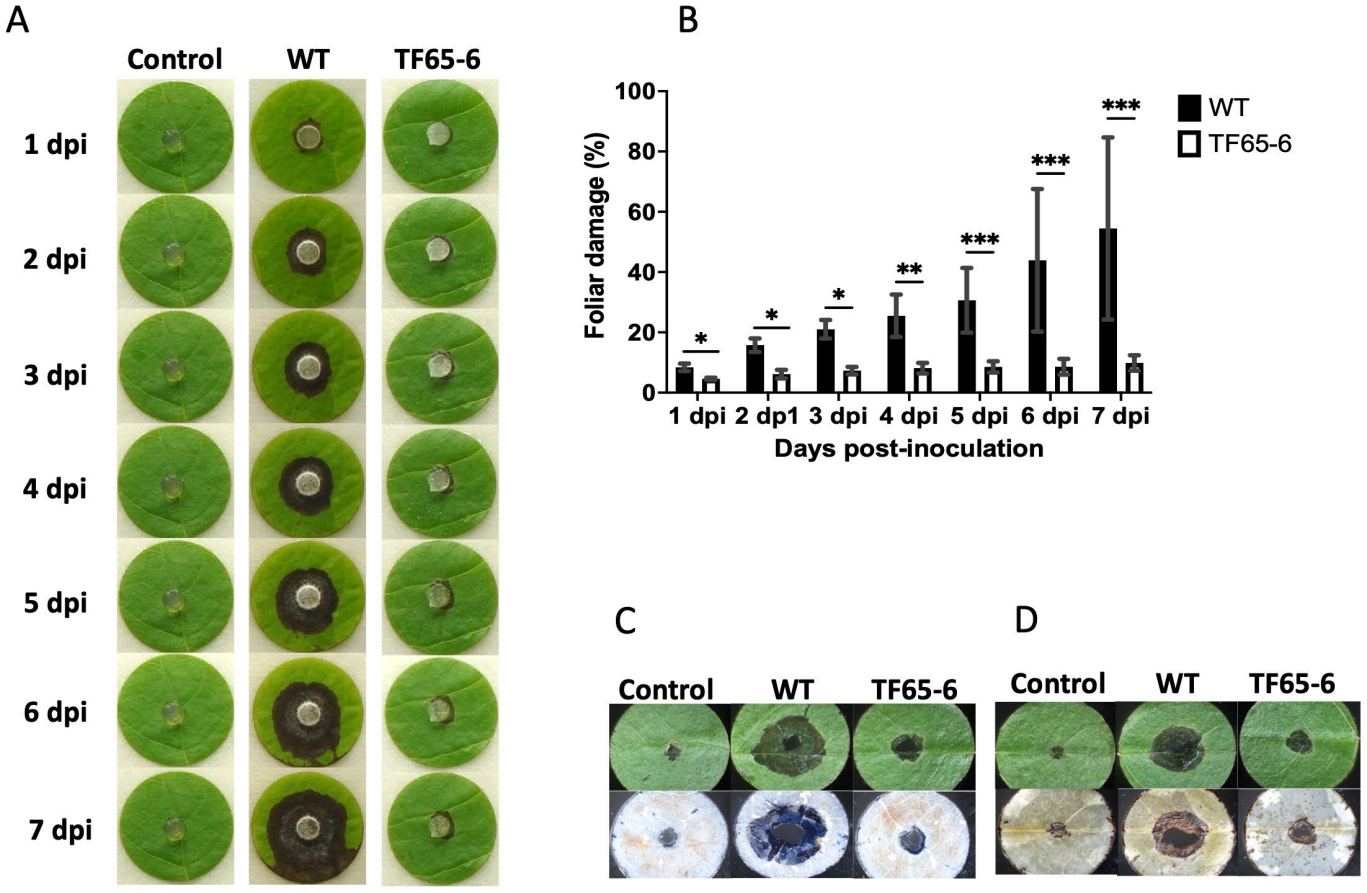
FspTF regulates pathogenesis in *Fusarium* sp. INECOL_BM-06. **(A)** Infection progression during 7 dpi in the foliar disk of *Populus nigra* inoculated with *Fusarium* sp. INECOL_BM-06 WT and TF65-6. **(B)** Progression of the percentage of damage of the foliar disk of *Populus nigra* inoculated with *Fusarium* sp. INECOL_BM-06 WT and TF65-6 during 7 dpi. **(C)** Plant cell death detection after 4 dpi with *Fusarium* sp. INECOL_BM-06 WT and TF65-6. Foliar disk showed before (upper panel) and after (down panel) trypan blue staining. **(D)** ROS (H_2_O_2_) detection after 4 dpi with *Fusarium* sp. INECOL_BM-06 WT and TF65-6. Foliar disk showed before (upper panel) and after (down panel) DAB staining. Bars in **(B)** are the average ± SD of ten technical replicates. (***value of *p*<0.001, **value of *p*<0.01, *value of *p*<0.05 by Two-way ANOVA with Bonferroni multiple comparison correction).

Since *Fusarium* sp. was reported as a potential phytopathogen vectored by *X. morigerus* (Carreras-Villaseñor et al., 2022), we investigated the role of FspTF in pathogenicity against the foliar disk of *Populus nigra* (Figure 4), *Coffea arabica*, *Citrus sinensis*, and *Citrus latifolia* (Supplementary Figure S4). The lesion in the plant tissue by the WT strain was characterized by the increasing necrosis and chlorosis area from the inoculation site along the time course tested. Interestingly, the mutant strain showed a significant negative impact on their infection capacity. Nevertheless, the impact was different among the plant species. In *P. nigra*, the edited strain provoked necrosis around the inoculation site, however the percentage of plant tissue damage at the end of the tested period was 54 and 9.8% by WT and TF65-6, respectively, denoting a clear difference in the infection progression (Figures 4A,B). The differences in the infection capacity were also noted during cell death and ROS detection in the plant tissue since the blue staining and the brown precipitate of DAB reacting with ROS were of lesser magnitude in foliar disks infected with TF65-6 (Figures 4C,D). In *C. arabica*, as in *P. nigra*, WT and TF65-6 established the infection process, but the progression was slower in the tissue infected by the mutant, denoted by the significant difference in the percentage of tissue damage along the tested period, being 17.7 and 14.3% at 7 dpi, respectively (Supplementary Figure S4). The progression of the infection by the mutant in the citrus species was slower until a certain time point; in *C. sinensis* the percentage of damage of the plant tissue was minor up to 2 dpi by TF65-6 (6.6%) in comparison by WT (9.3%), then the damaged area was comparable among the strains. In *C. latifolia* the damage in plant tissue by all the strains was comparable from 5 dpi, before that, the foliar disk inoculated with the mutant strains developed minor symptoms compared to those inoculated with the WT (Supplementary Figure S4).

**Figure 5:**
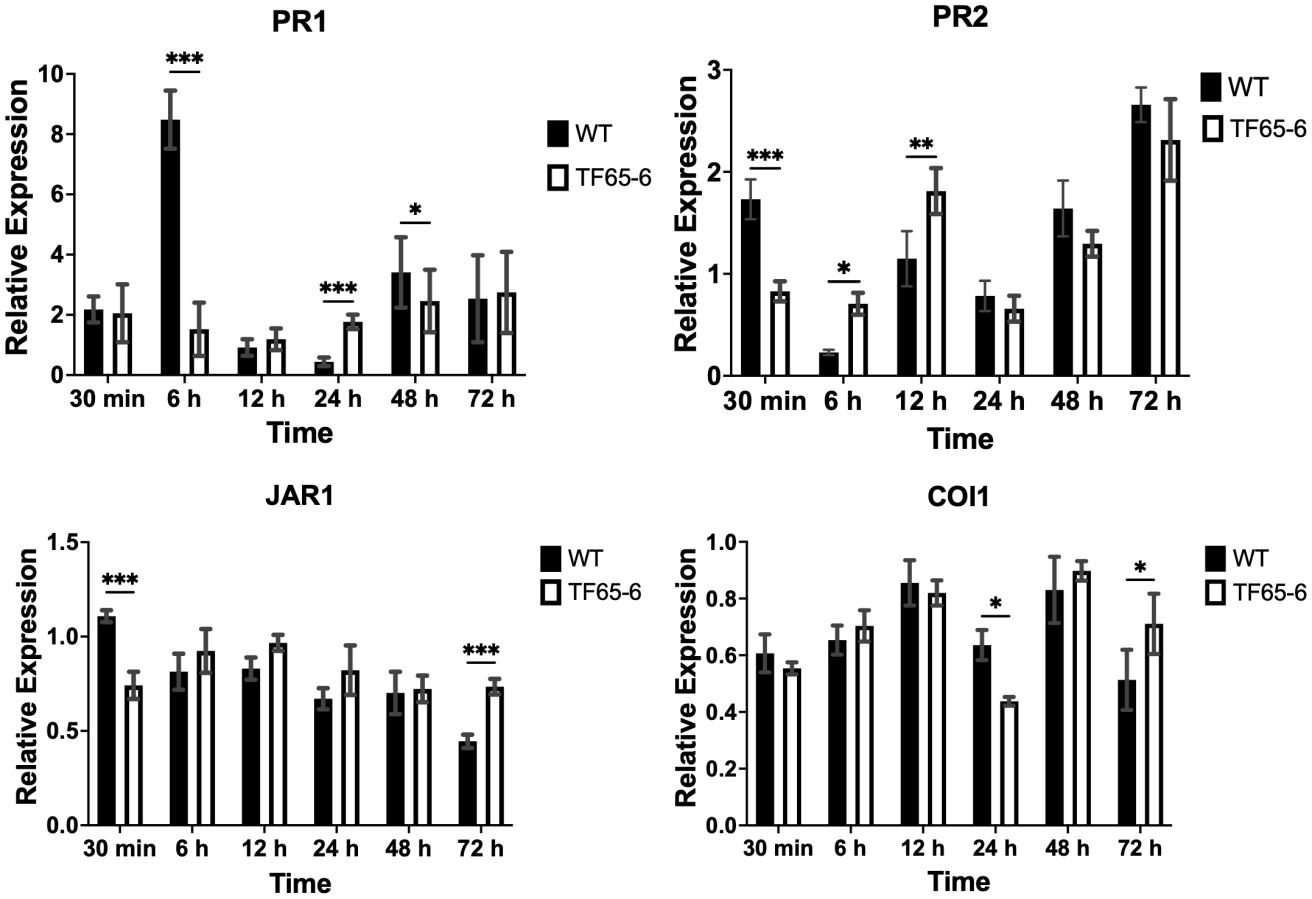
Defense response of *Populus nigra* infected by *Fusarium* sp. WT and TF65-6. Relative expression of *PR1* and *PR2* in the SA signal transduction pathway, and *JAR1* and *COI1* in the JA signal transduction pathway of *Populus nigra* inoculated with *Fusarium* sp. INECOL_BM-06 WT and TF65-6. Expression is relative to *UBQ* expression and control condition. Bars are average ± SD of three technical replicates. (***value of *p*<0.001, **value of *p*<0.01, *value of *p*<0.05 by Two-way ANOVA with Bonferroni multiple comparison correction).

Later, we evaluated the defense system of *P. nigra* in response to infection with *Fusarium* sp. WT and TF65-6. Interestingly, the expression of *PR1* and *PR2* as marker genes of the salicylic acid (SA) pathway and *JAR1* and *COI1* involved in the jasmonic acid (JA) pathway in response to TF65-6 was different in comparison to WT, mainly showing a delay and less intensity in the response (Figure 5). Specifically, the expression profile of *PR1* in response to the WT implied an induction from 30 min (2.2 fold), with a major increase in the expression level, 6.5 fold, at six hpi; then, repression was observed reaching a basal level at 12 hpi and the repression increased at 24 hpi (0.4 fold), with a tendency toward induction from 48 hpi with a 3.4 fold increase. In contrast, in the *PR1* expression profile in response to TF65-6, an induction was observed at 30 min (2.1fold, similar in response to the WT), followed by a tendency to repress the expression up to 12 hpi (1.2 fold), and, at 24 hpi (1.8 fold), a trend toward induction was observed until reaching a similar expression in response to both strains at 72 hpi (2.7 fold). The expression profile of *PR2* in response to the WT strain implies “transcriptional waves” of induction-repression of expression up to 24 hpi, with the higher induction at 30 min (1.7 fold), and at 6 hpi the expression was the lowest (0.2 fold). From 48 hpi, the trend was toward induction of expression, reaching the highest level at 72 hpi (2.7 fold); however, the plant infected with TF65-6 did not seem to respond until 12 hpi, where induction of *PR2* expression was observed (1.8 fold) with subsequent repression at 24 hpi (0.7 fold), followed by a tendency toward induction from 48 hpi onwards, reaching an induction of 2.3 fold at 72 hpi, as well as the response against the WT strain (Figure 5). Regarding the expression of marker genes for the JA pathway, *JAR1* was repressed in the interaction with the WT, with greater repression being observed at 72 hpi (0.4 fold). On the other hand, there seemed to be no response against the TF65-6 mutant strain or with a slight tendency to repression, to a lesser extent than against the WT strain, especially at 72 hpi (0.7fold). In relation to the expression of the *COI1* gene, it was observed that for both strains, the response was the repression of the expression, presenting practically the same levels in response to both strains, except at 24 hpi, where the repression was more significant in the strain TF65-6 (0.4 fold and 0.6 for WT) (Figure 5).

#### 3.4.3. Secondary metabolism is controlled by FspTF

**Figure 6:**
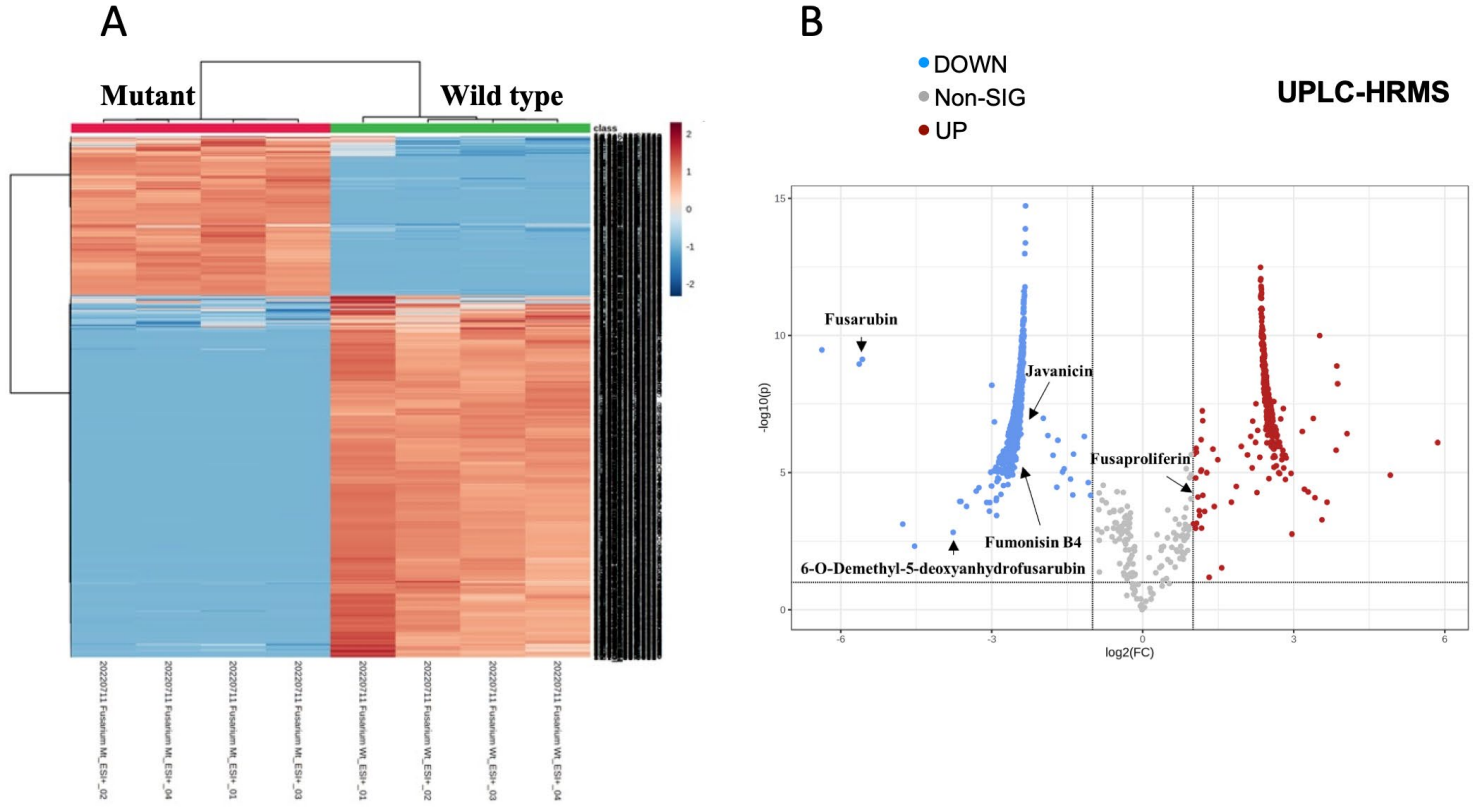
Metabolomic profile of *Fusarium* sp. TF65-6. **(A)** Heatmap of ESI^+^ and ESI^−^ rt-m/z signals from *Fusarium* sp. INECOL_BM-06 WT and TF65-6. **(B)** Volcano plot representing the differentially accumulated metabolites in TF65-6. Some tentatively identified compounds are shown.

We performed a comparative untargeted metabolomic analysis between the mycelium of the WT and TF65-6 obtained after 7 days of growth in PDA (Figure 6). We detected 1,694 spectrometric features (retention time-mass/charge ratios; rt-m/z) in both ESI^+^ and ESI^−^ modes in the dataset. A differential accumulation analysis made it evident that fewer rt-m/z signals were accumulated in the TF65-6 (Figure 6A), with 351 rt-m/z signals over-accumulated, and 833 rt-m/z signals down-accumulated in TF65-6 compared to WT, and 465 rt-m/z signals were not statistically different (Figure 6B). Among the rt-m/z signals detected and over-accumulated in TF65-6, fusaproliferin, classified as an emerging *Fusariu*m mycotoxin (Gruber-Dorninger et al., 2017; Ćeranić et al., 2021), and some lipids, such as linolenic and palmitic acids (Figure 6B; Table 1) were putatively identified. Among those that were down-accumulated were fusarubin, 6-O-demethyl-5-deoxyanhydrofusarubin, and javanicin, which are pigmented polyketides produced by different *Fusarium* species (Studt et al., 2012; Kristensen et al., 2021) and fumonisin B4, the less common fumonisin of the B series (Awuchi et al., 2021; Ekwomadu et al., 2021) tentatively identified (Figure 6B; Table 1).

**Table 1:** Metabolites differentially accumulated in *Fusarium* sp. TF65-6 tentatively identified.

RT (min)	*m/z* (Da)	Fold Change	Mass error (ppm)	Adduct	Metabolite
6.78	305.0657	-5.57	-1.31	[M-H]^-^	Fusarubin
7.85	259.0615	-3.76	3.47	[M+H]^+^	6-O-Demethyl-5-deoxyanhydrofusarubin
11.91	434.0237	-2.76	-1.33	[M+Na]^+^	Deoxyadenosine diphosphate
7.92	690.4049	-2.55	-2.32	[M+H]^+^	Fumonisin B4
10.79	287.063	-2.53	-4.98	[M+H]^+^	Glycineamideribotide
8.27	313.0348	-2.52	3.2	[M-H_2_O-H]^-^	Deoxyinosine monophosphate
7.99	289.071	-2.5	-0.69	[M-H]^-^	Javanicin
7.76	268.0368	-2.49	-2.56	[M-H_2_O-H]^-^	Indoleglycerol phosphate
14.82	301.216	-2.44	-2.51	[M-H]^-^	Eicosapentaenoic acid
5.74	165.055	-2.42	-1.03	[M+H]^+^	Phenylpyruvic acid
8.73	259.0696	-2.41	0.47	[M+Na]^+^	L-Formylkynurenine
11.69	121.0651	-2.41	-1.97	[M+H]^+^	Phenylacetaldehyde
6.27	229.0482	-2.41	2.11	[M+H]^+^	Mevalonic acid-5P
13.08	279.2322	1.06	-0.73	[M+H]^+^	Linolenic acid
13.25	445.2951	1.12	-0.67	[M+H]^+^	Fusaproliferin
14	255.2323	2.43	-0.41	[M-H]^-^	Palmitic acid
12.86	300.2907	2.45	1.49	[M+H]^+^	Sphingosine/3-Dehydrosphinganine
13.3	279.2321	2.56	-1.09	[M+H]^+^	Linoleic acid

RT, Retention time in minutes.

#### 3.4.4. Overview of the transcriptome analysis in TF65-6

**Figure 7:**
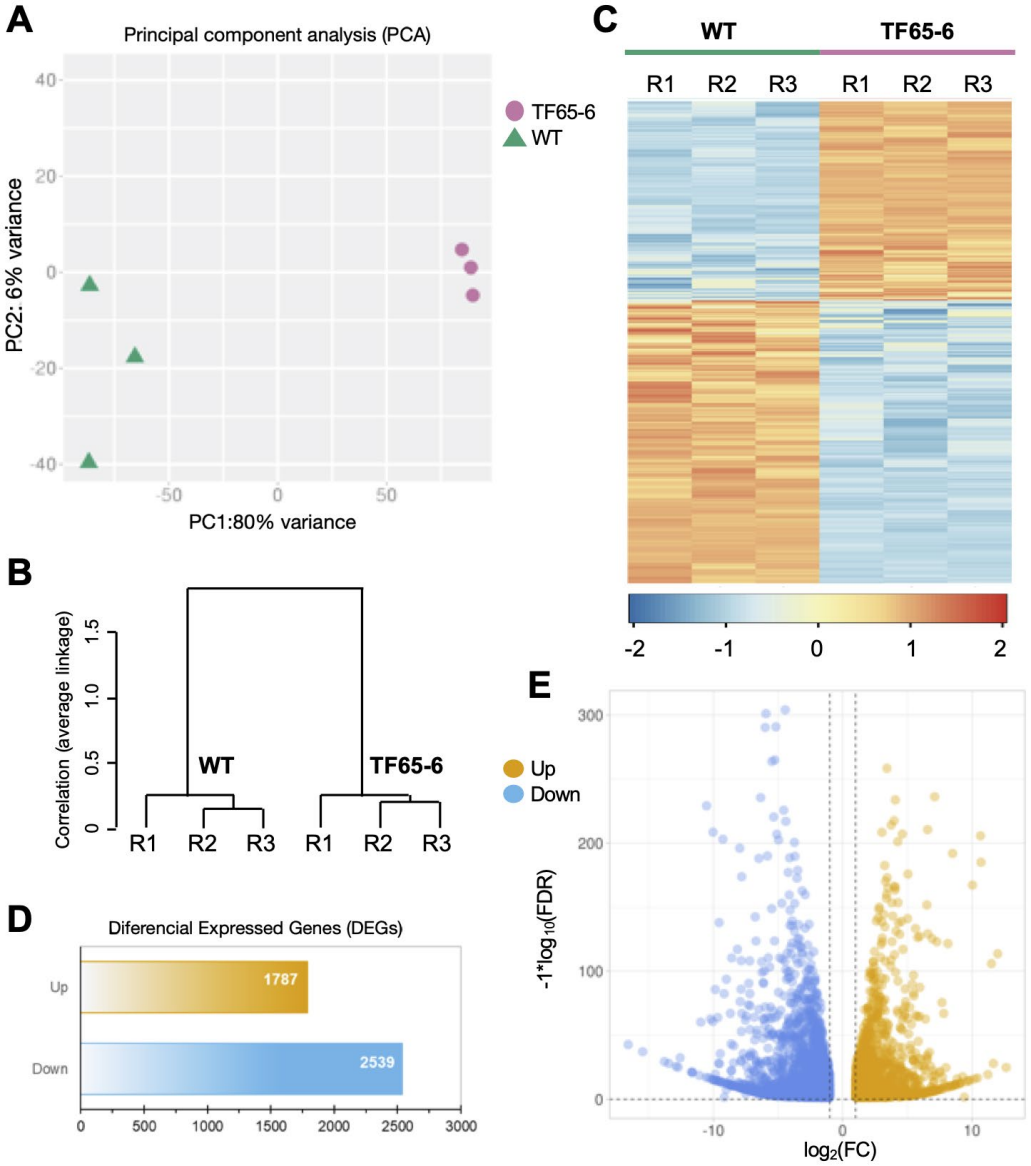
Differential gene expression in *Fusarium* sp. INECOL_BM-06 TF65-6. **(A)** Principal component analysis (PCA) plot displaying all six generated and used libraries in the presented study (three independent biological replicates (R1-R3) generated for WT and TF65-6, respectively). PCA was applied to the transcripts per million (TPM) values. **(B)** Hierarchical clustering tree of both WT and TF65-6 strains; the tree was performed using the maximum TPM values estimated for 75% of the genes and considering their independent biological replicates. The correlation (average linkage) for the clades is shown at the left of the tree. **(C)** Heat map displaying expression levels from WT and the TF65-6. Each column in the heatmap corresponds to each of the biological replicates (R1-R3). The cluster color code is at the bottom of the heatmap. **(D)** Bars and **(E)** volcano plot representation of the differentially expressed genes identified in the TF65-6 mutant (a pairwise comparison of TF65-6 vs. WT). The yellow and blue bars **(D)**, or dots **(E)**, indicate up- and down-regulated genes, respectively. [log_2_ fold change (FC) ± 1, *value of p* (FDR) ≤ 0.05].

We used high-throughput sequencing to examine the genome-wide transcript profiles of the WT and TF65-6 mycelium samples collected from colonies grown on PDA for 7 days. The raw sequencing data consist of 13 to 18 million reads for each library, suggesting good coverage and sequencing depth. After the quality analysis, a total of 90,642,783 high-quality (HQ) paired-end reads were obtained (Supplementary Table S4). These HQ reads were mapped against the *Fusarium* sp. INECOL_BM-06 genome and quantified (Supplementary Table S5). To carry out the differential expression analysis (Figure 7), a principal component analysis (PCA), using the estimated TPM values, was performed to detect the major sources of variance underlying the biological replicates (R1-R3) of each strain. The two-dimensional PC plot with the first two principal components (PC1 and PC2) best illustrated the variance among the expression profiles, with a proportion of explained variance of 80 and 6%, respectively (Figure 7A). The plot, together with a hierarchical clustering tree of the six libraries (Figure 7B), indicated that the biological replicates have high reproducibility. Based on these results, a pairwise comparison (TF65-6 vs. WT) was performed to identify differentially expressed genes (DEG). In total, 4,326 genes were encountered with differential expression of at least two-fold (Log2FC = ±1) and an adjusted significant value of p of ≤0.05 (Figures 7C–E; Supplementary Tables S6–S7), and as expected, the expression profile among the biological replicates was comparable (Figure 7C). From the differential genes, 1,787 genes were induced, and 2,539 genes were repressed in TF65-6 relative to WT (Figure 7D), most of them with Log2FC = ±5 (Figure 7E; Supplementary Table S7).

**Figure 8:**
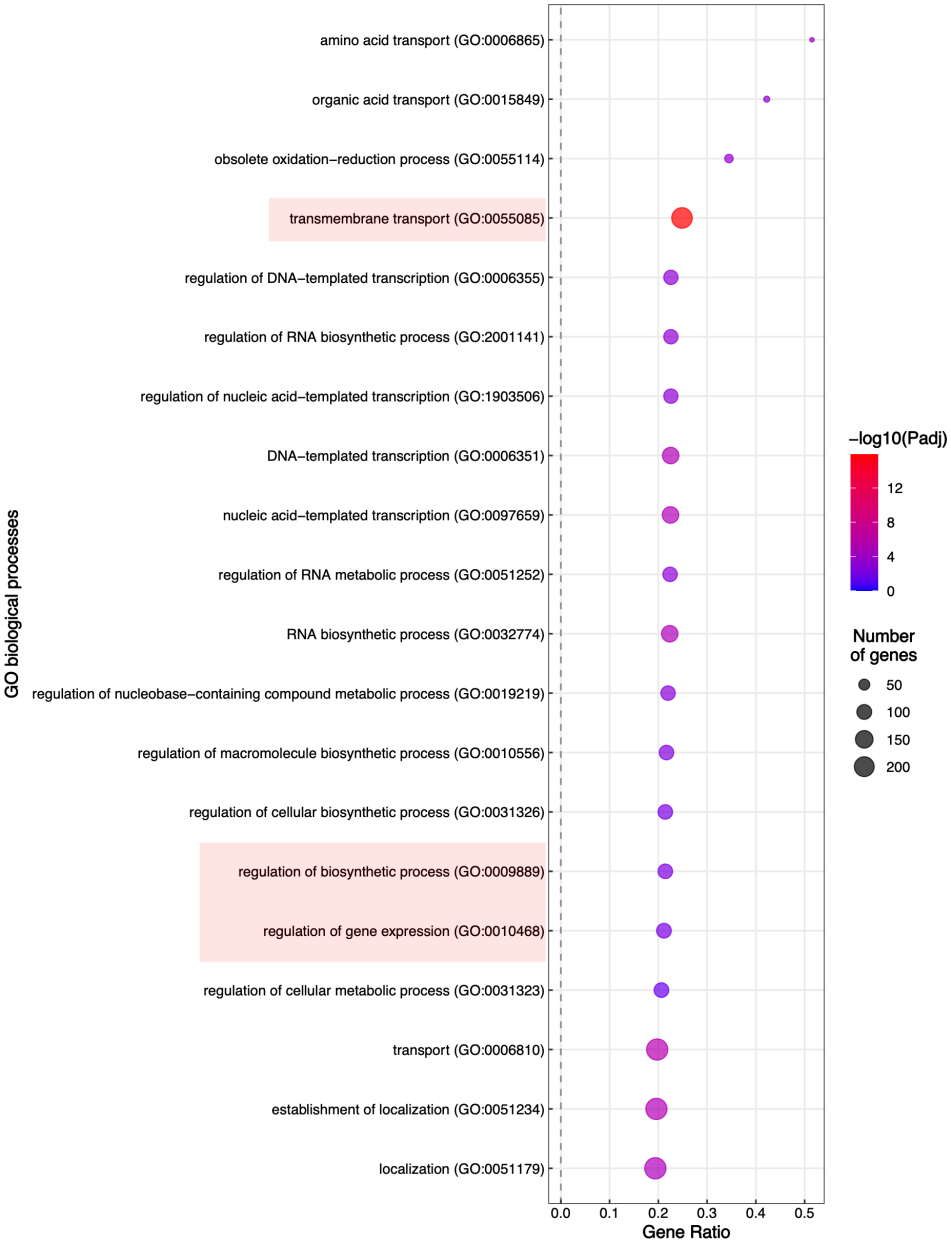
Enrichment of the Biological Process Gene Ontology (GO) terms. Significantly enriched GO terms are shown; highlighted are the GO terms to which belong most of the DEGs in TF65-6.

To evaluate which processes were regulated by FspTF, we performed an enrichment analysis of gene ontology (GO) terms and KEGG metabolic pathways. In all, 12 molecular functions (MF) terms, 4 cellular components (CC) terms (Supplementary Table S8), and 19 biological processes (BP) terms (Figure 8) were significantly enriched by 1,194 of the DEGs (27.6% of the total) (Supplementary Table S8). Specifically, within the MF category, the DEGs were enriched in four ‘parent’ terms: transporter activity (GO:0005215), oxidoreductase activity (GO:0016491), transcription regulator activity (GO:0140110), and metal ion binding (GO:0046872). For the CC category, DEGs were enriched in the membrane (GO:0016020) term (Supplementary Table S2); finally, in the BP category, most of the DEGs belonged to three enriched ‘parent’ terms: regulation of biosynthetic process (GO:0009889), regulation of gene expression (GO: 0010468), and the transmembrane transport (GO: 005085; Figure 8). On the other hand, the enriched KEGG term was metabolic pathways (KEGG: 01100; Supplementary Table S8).

##### 3.4.4.1. Regulation of transmembrane transport: antifungal resistance

**Figure 9:**
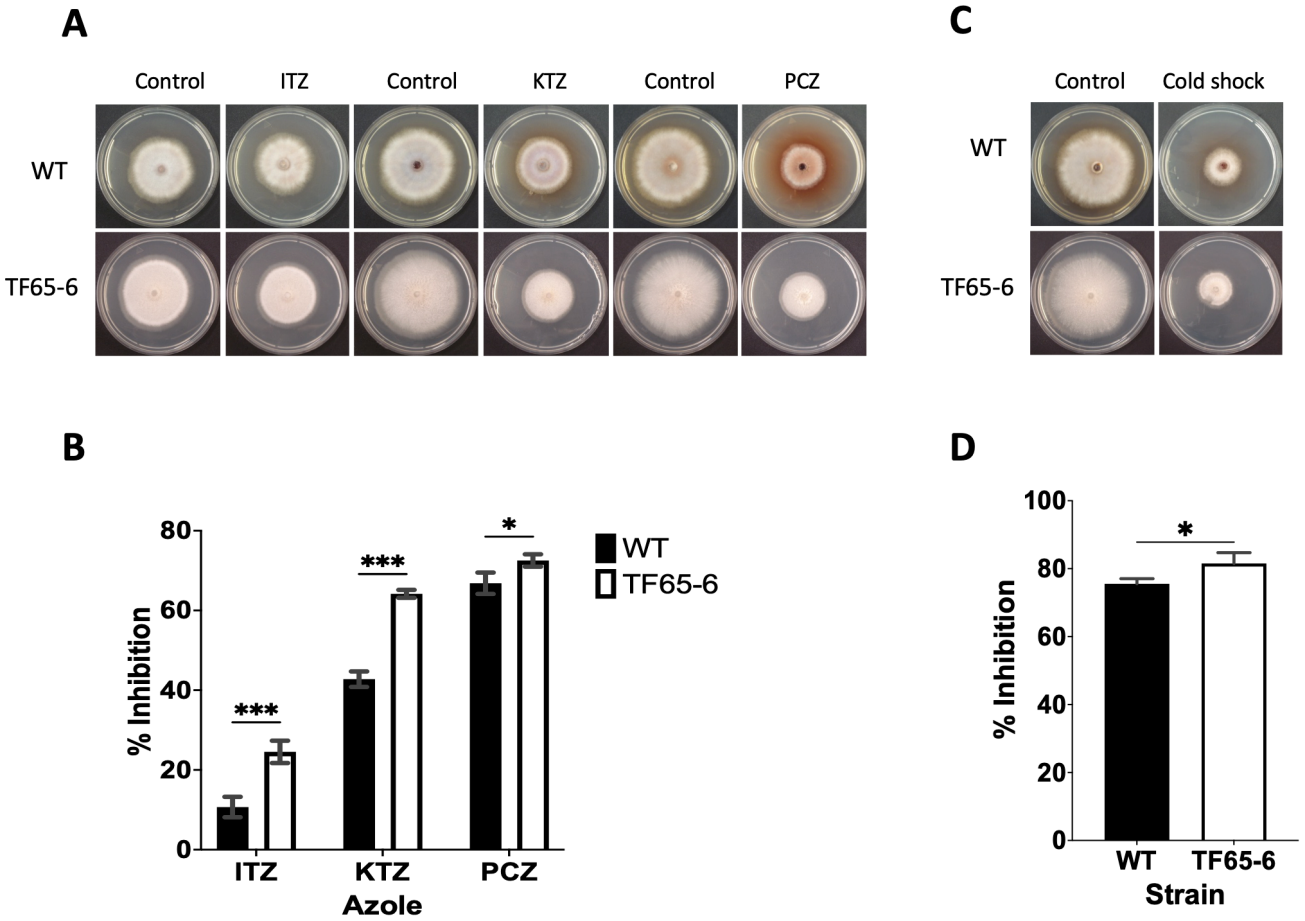
FspTF regulates the susceptibility to azole fungicides and cold shock in *Fusarium* sp. INECOL_BM-06. **(A)** Growth of *Fusarium* sp. INECOL_BM-06 WT and TF65-6 in PDA supplemented with different azole fungicides. **(B)** Mycelial growth inhibition of *Fusarium* sp. INECOL_BM-06 WT and TF65-6 grown in PDA amended with different azole fungicides. ITZ: Itraconazole 64 μg/mL. KTZ: Ketoconazole 16 μg/mL. PCZ: Propiconazole 4 μg/mL. Data were obtained at 7 dpi. **(C)** Growth of *Fusarium* sp. INECOL_BM-06 WT and TF65-6 in PDA after cold shock. **(D)** Mycelial growth inhibition of INECOL_BM-06 WT and TF65-6 grown in PDA after cold shock. Bars are the average ± SD of three technical replicates. (***value of *p*<0.001, **value of *p*<0.01, *value of *p*<0.05 by Two-way ANOVA with Bonferroni multiple comparison correction. In D *value of *p*<0.01 by *t*-test).

In the transmembrane transport category (GO: 0055085), there were 272 DEGs classified (Supplementary Table S8), including transporters whose probable substrates are diverse such as carbon and nitrogen sources, vitamins, nucleobases, inorganic compounds as cations (K^+^ and Na^+^), phosphates, heavy metals, and xenobiotics (Supplementary Table S8). We identified 151 enriched DEGs encoding major facilitator superfamily (MFS) transporters; from those, 88 and 63 DEGs were induced and repressed, respectively. The most induced MSF transporter (ID: g09286, FC: 10.2) was predicted to be an allantoate permease and, notably, the most repressed was the homologous of *FLR1* (ID: g05833, FC: −8.6) involved in conferring resistance to a wide range of chemical to yeast (Sá-Correia et al., 2009; Supplementary Table S8). In addition, ATP-binding cassette (ABC) transport pumps were encoded by 16 enriched DEGs, 5 DEGs were upregulated, and 11 DEGs were downregulated. The most induced DEG (ID: g06701, FC: 3.3) was the homologous of *STE6* required for the export of the α-factor. Within the repressed genes, there were DEGs homologous of ABC-transporters involved in the pleiotropic drug resistance (Kovalchuk and Driessen, 2010; Víglaš and Olejníková, 2021), being *PDR15* (ID: g07623, FC: −6.4) the most downregulated (Supplementary Table S8). Given these data, WT and TF65-6 strains were treated with itraconazole (ITZ) 64 μg/mL, ketoconazole (KTZ) 16 μg/mL, and propiconazole (PCZ) 4 μg/mL, with TF65-6 being 2.3, 1.5, and 1.1fold more sensitive than WT to the azole compound, respectively. Furthermore, ITZ was the fungicide with more effect in the mutant (Figures 9A,B).

Regarding nutrient transport, we identified 13 DEGs homologous to hexose transporters (HXT), and most of them were upregulated (11 DEGs). A homologous of *HXT9* (ID: g15700, FC: 6.08) was the most induced, and a homologous of *HXT2* (ID: g01538, FC: −2.89), the high-affinity transporter, the most repressed. In addition, transporters of other sugars, such as α-glucosides, like maltose, were also induced (ID: g02552, FC: 6.1; ID: g03953, FC: 3.4; ID: g02415, FC: 2.9; ID: g00875, FC: 2.1 and ID: g12844, FC: 1.5; Supplementary Table S8). Besides, we identified that *Fusarium* sp. TF65-6 overexpressed 19 genes that were enriched in the amino acid transport (GO: 0006865) category, most of them were members of the APC (amino acid-polyamine-organocation), such as *GAP1* (ID: g15708 FC: 6.3), *PUT4* (ID: g07982 FC: 1.7), *DIP5* (ID: g13826 FC: 1.6), and *AGP3* (ID g:05215 FC: 1.6; Bianchi et al., 2019; Supplementary Table S8). Finally, we detected that TF65-6 expressed DEGs homologous to genes related to the transporter of essential macronutrients and micronutrients such as potassium, phosphate, zinc, copper, and iron (Supplementary Table S8).

##### 3.4.4.2. Regulation of gene expression of metabolic pathways

The DEGs were involved in diverse metabolic pathways (KEGG: 01100), and those genes enclosed in the regulation of biosynthetic process (GO:0009889) category were also part of the regulation of gene expression (GO:0010468) category, suggesting that those genes might be involved in the transcriptional control of metabolic processes (Supplementary Table S8). We identified *Fusarium* sp. homologous genes that encode TFs or transcriptional regulators of diverse metabolic pathways. For example, homologous genes involved in the metabolism of preferred and secondary nitrogen sources, such as *UGA3* (ID: g13659, FC: 2.7), necessary for the utilization of GABA (γ-aminobutyric acid) (Cardillo et al., 2011; Sylvain et al., 2011), *nit-4* (ID: g05829 FC: −1.01), the specific TF in the nitrogen circuit of *N. crassa* (Yuan et al., 1991; Huberman et al., 2021); *ARG81* (ID: g11156 FC: −1.2) component of the ArgR-Mcm1complex involved in the control of arginine metabolism (Dubois and Messenguy, 1985; Amar et al., 2000), *LYS14* (ID: g07676 FC: −3.8), a transcriptional activator of genes for lysine biosynthesis (Feller et al., 1994) and *ARO80* (ID: g15512 FC: −1.9), the TF that activates the expression of genes involved in the catabolism of aromatic amino acids (Lee and Hahn, 2013). We also identified TFs involved in the metabolism of carbon substrates such as *CAT8* (ID: g09824 FC: −1.5), a TF required for the utilization of non-fermentative carbon sources (Hedges et al., 1995; Haurie et al., 2001), *MAL13* (ID: g06159 FC: −1.1) and *MAL33* (ID: g09280 FC: −1.09), TFs components of the *MAL1* and *MAL3* locus, respectively, required for the fermentation of maltose (Charron et al., 1986; Orikasa et al., 2018) and *GAL4* (ID g05617 FC: −4.4), the key TF for the glucose and galactose catabolism (Choudhury and Whiteway, 2018). With respect to lipid metabolism, we identified *ASG1* (ID: g01923 FC: −6.5), a TF involved in the activation of the ß-oxidation pathway (Jansuriyakul et al., 2016). In addition, we also identified *cft1α* (ID: g02375 FC: −2.2) and *cft1β* (ID: g13796 FC: −3.7), which are TFs that are required for the expression of cutinases in *F. solani* (Li et al., 2002), and *UPC2* (ID: g15625 FC: −4.4) and *ECM22* (ID: g02029 FC:-3.2), which are TFs required for the ergosterol biosynthesis (Joshua and Höfken, 2017; Jordá and Puig, 2020), among others TFs with roles in purine/pyrimidine metabolism and regulators of transcription and translation (Supplementary Table S8).

##### 3.4.4.3. Regulation of secondary metabolism: pigments and mycotoxins

Since the evident differences in pigment production between mutant and WT strains and the metabolomic results (Figures 2, 6; Table 1), we delved into the analysis of genes related to secondary metabolism. In this sense, genes predicted to encode polyketides synthases (PKS) and non-ribosomal peptide synthases (NRPS) are downregulated in mutants, such as *PGL1* (ID: g05609 FC: -6.7), *BIK1* (ID: g8257 FC: −5.1), and *FUB1* (ID: g07760, FC: −3.89), that are required for perithecium pigment, bikaverin and fusaric acid production in *F. verticilloides*, respectively (Table 2). In addition, homologous genes of some FSR cluster genes, required for fusarubin synthesis in *F. fujikuroi* (Studt et al., 2012), were encountered as downregulated, and homologous genes of FUS cluster genes, required for fusarin production in *F. verticilloides* (Brown et al., 2012) were identified as repressed. In addition, *FUM 13* (ID: g01406, FC: −1.8), a short-chain dehydrogenase/reductase required for the fumonisin biosynthesis in *F. verticilloides* (Butchko et al., 2003), was also repressed (Table 2).

**Table 2:** Differential genes in *Fusarium* sp. TF65-6 related to pigments and mycotoxins, putative effectors or virulence factors, and lipid components of the cell membrane.

Gene-ID	Fold Change	Gene	Function
Mycotoxin: Fusarin cluster genes (*FUS*)			
g13087	-5.65	*FUS1*	Fusarin C cluster-polyketide synthase/NRPS
g13080	-11.09	*FUS2*	Fusarin C cluster-hydrolase
g13081	-9.54	*FUS3*	fusarin C cluster-translation Elongation factor
g13082	-7.88	*FUS4*	Fusarin C cluster-peptidase
g13101	-6.92	*FUS6*	Fusarin C cluster-transporter
g13091	-9.95	*FUS7*	Fusarin C cluster-oxidoreductase
g13097	-12.74	*FUS8*	Fusarin C cluster-cytochrome P450
g13098	-10.04	*FUS9*	Fusarin C cluster-methyltransferase
Pigment: Fusarubin cluster genes (*FSR*)			
g05609	-6.72	*FSR1/PGL1*	Fusarubin cluster-polyketide synthase
g05588	-7.18	*FSR2/PGL3*	Fusarubin cluster-methyltransferase
g05615	-4.61	*FSR3/PGL4*	Fusarubin cluster-oxidoreductase
g05606	-5.07	*FSR4/PGL5*	Fusarubin cluster-dehydrogenase
Mycotoxin and Pigments: Genes of other biosynthetic clusters			
g08257	-5.12	*BIK1*	PKS for bikaverin pigment synthesis
g07760	-3.90	*FUB1*	PKS for fusaric acid synthesis
g01406	-1.83	*FUM13*	Fumonisin cluster-short-chain Dehydrogenase
Non-Ribosomal Peptide Synthetases (NRPS)			
g01554	3.45	-	NRPS
g11025	2.14	-	NRPS
g12634	-1.00	-	NRPS
g08990	-1.35	-	NRPS
g07840	-1.68	-	NRPS
g10481	-2.11	-	NRPS
g12060	-2.48	-	NRPS
g11043	-5.58	-	NRPS
Putative effectors or virulence factors			
g11267	7.816	Adhesin-like	---
g00036	7.528	Necrosis inducing protein-domain-containing protein	Apoplastic effector
g10639	7.142	Ribonuclease/ribotoxin	Apoplastic effector
g04647	6.844	Lysozyme-like domain-containing protein	Cytoplasmic/apoplastic effector
g02445	6.729	LysM domain-containing protein	---
g00871	5.982	Secreted protein	Apoplastic effector
g00991	3.289	CFEM domain-containing protein	---
g00949	2.679	Extracellular serine-rich protein	---
g00944	1.283	Necrosis inducing protein-domain-containing protein	Apoplastic effector
Cell membrane components: Phospholipids biosynthesis			
g04816	-1.09	*CDS1*	Phosphatidate cytidylyltransferase
g07564	-3.71	*EK1*	Ethanolamine kinase
g06068	-3.77	-	phosphatidylserine decarboxylase
g05488	-6.65	-	phosphatidylserine decarboxylase
Cell membrane components: Sphingolipids biosynthesis			
g01424	-1.11	*TSC-10*	3-ketodihydrosphingosine reductase
g04910	-1.30	*LCB4*	Sphingoid long-chain base kinase
g04373	-1.86	*SUR2*	Sphinganine hydroxylase
g11362	-2.83	*LCB2*	Serine C-palmitoyltransferase
Cell membrane components: Ergosterol biosynthesis			
g06230	1.99	*ERG25*	C-4 methylsterol monooxygenase
g08073	1.95	*ERG6*	C24-sterol methyltransferase
g14567	1.30	*ERG11*	Lanosterol 14 alfa-demethylase
g08287	1.21	*ERG1*	Squalene epoxidase
g09701	1.15	*ERG4*	C-24 Sterol reductase
g05849	-1.16	*ERG5*	C-22 Sterol desaturase
g11629	-1.19	*ERG28*	Endoplasmic reticulum membrane protein
g02954	-1.43	*ERG7*	Lanosterol synthase
g08093	-7.23	*ERG10*	Acetyl-CoA C-acyltransferase

##### 3.4.4.4. Virulence

*Fusarium* sp. INECOL_BM-06 is reported as a phytopathogen (Carreras-Villaseñor et al., 2022), and the progression of the infection process is compromised in *Fusarium* sp. TF65-6 (Figure 4; Supplementary Figure S4). In this context, we searched for genes with possible roles in pathogenicity, with emphasis on those that encode secreted hydrolytic enzymes and virulence factors or effectors. We focused on the enzymes that target cuticle and plant cell walls, the first barriers of pathogen invasion, and proteases (Supplementary Table S9).

Among the carbohydrate esterases (CE) involved in lipid degradation, we encountered nine DEGs. A cutinase-domain-containing protein was induced (ID: g05189, FC: 2.23), and different secreted lipases were expressed differentially in TF65-6, from which the homologous of *FGL1* (ID: g06428, FC: −5.85), a lipase belonging to Abhydrolase superfamily (cl21494) and reported as a virulence factor in *F. graminearum* (Voigt et al., 2005), was the most repressed (Supplementary Table S9).

Moreover, in TF65-6, some DEGs encode glycoside hydrolases (GH) from different families that degrade the cellulose, hemicellulose, and pectin, components of the primary plant cell wall, as well as oxidases that degrade lignin, the secondary cell wall (Supplementary Table S9). There are 26 DEGs predicted to be involved in cellulose degradation. The highest induced gene encodes weak endoglucanase (ID: g08682, FC: 5.31) belonging to the lytic polysaccharide monooxygenase (LPMO) auxiliary superfamily (cl41755), and the most repressed gene was predicted to be a β-glucosidase (ID: g06562, FC: −5.72) of the BglX superfamily (cl34276). Most of the induced genes belonged to the GH6 (cl38036), GH7 (cl21662), GH12 (cl03302), GH45 (cl03405), and GH61 (cl04076) superfamilies, and among the repressed genes, there was a member of the GH3 (cl38063) superfamily (Supplementary Table S9). There were seven DEGs predicted to be GH that degrade hemicellulose; all were induced, being an endo-1,4-β-xylanase (ID: g00072, FC: 4.74), the most upregulated. These GHs belonged to the GH10 (cl23725), GH11 (cl08271), GH43, and GH62 (cl14647) superfamilies (Supplementary Table S9). Regarding enzymes involved in pectin degradation, there were 18 DEGs and most of them were induced (16 DEGs). Interestingly, in this category were the secreted hydrolytic enzymes that showed the highest induction and the most repressed, a pectinesterase (ID: g02162, FC: 7.67) (cl29893) and a pectin lyase fold-protein (ID: g01523, FC: −5.94), respectively; besides there were members of the GH28 (cl37622) and GH114 (cl45921) superfamilies (Supplementary Table S9). Concerning genes involved in lignin degradation, there were four DEGs; all predicted to be laccases belonging to the cupredoxin superfamily (cl9115); in this category were the genes with the highest fold change related to plant cell wall degradation, g02419 (FC: 8.72) and g01716 (FC: −8.92; Supplementary Table S9).

With respect to secreted proteases, we found 21 DEGs in TF65-6. From the eight induced genes, four (g01797, g07709, g15367, and g01991) were serine-proteases S8 (cd07489 and cd04077) and two (g05209 and g11513) were metalloproteases M35 (cl03449) and M28 (cd03876), respectively, being the last the protease most induced (FC: 5.42; Supplementary Table S9). Among the 13 repressed proteases, the most repressed (ID: g13082, FC: −7.88) was an aspartic protease (cd05471; Supplementary Table S9).

Regarding genes that encode possible effectors or virulence factors, TF65-6 expressed DEGs that encoded a necrosis-inducing protein, ribonuclease/ribotoxin, LysM protein, and CFEM protein, among others, most of them induced (Table 2). Interestingly, some genes involved in pectin degradation were predicted to be apoplastic effectors, including the most repressed gene, the pectin lyase fold-protein (ID: g01523, FC: −5.94; Supplementary Table S9).

##### 3.4.4.5. Regulation of lipid metabolism: cell membrane

We recognized repressed genes involved in the biosynthesis of the lipid components of the cell membrane, such as glycerophospholipids, sphingolipids, and sterols (Table 2). For example, the homologous of *CDS1* (ID: g04816, FC: −1.09), which catalyzes the critical enzymatic reaction in the biosynthesis of all major phospholipids for producing the liponucleotide precursor, CDP-diacylglycerol (CDP-DAG; Sant et al., 2016). In addition, the most repressed gene involved in the biosynthesis of sphingolipids was the homologous of *LCB2* (ID: g11362, FC: −2.83), a serine palmitoyltransferase responsible for the first committed step of their biosynthetic pathway (Fabri et al., 2020). Worth mentioning is the ergosterol biosynthetic pathway. We identified 10 DEGs that were homologous to those involved in biosynthesis. Homologous to *ERG10*, the acetyl-CoA C-acyltransferase, which mediates the first step for the synthesis of the intermediate mevalonate, was the most repressed (ID: g08093, FC: −7.23). Genes involved in the late biosynthetic steps were deregulated; considering the two rate-limiting steps, *ERG1* and *ERG11* were induced, and genes that encode last enzymes of the pathway, suggested to display low substrate affinities, such as *ERG6* and *ERG4*, were induced, conversely, *ERG5* was repressed (Table 2; Supplementary Figure S5) (Jordá and Puig, 2020). Together these data suggest that the composition of the cell membrane may be altered, and because the changes in membrane composition might be necessary for cold stress response (Aguilera et al., 2007), we evaluated the growth phenotype of WT and TF65-6 by subjecting both strains to cold stress (4°C) during 72 h, and then resumed their growth at 25°C. We found that the mutant showed 6% more growth inhibition in comparison with the WT data, a discrete but statically significant difference (Figures 9C,D) denoting that TF65-6 was less tolerant to this stress than WT.

##### 3.4.4.6. Stress response: oxidative stress and detoxification.

Because Fsp*tf* expression was induced during oxidative stress and the edited strain showed discrete but significantly more sensitivity to oxidative stress, we searched the expression profile of genes related to scavenging mechanisms of ROS (Supplementary Table S10). Here, we found that TF65-6 repressed the expression of the homologous of the glutathione reductase *GRL1* (ID: g00990, FC: −1.35), glutathione peroxidase *HYR1* (ID: g03050, FC:-2.78), thioredoxin *TRX1* (ID: g08764), glutaredoxin *GRX2* (ID: g06203, FC: −2.2), superoxide dismutase 2 *SOD2* (ID: g12147, FC: −2.55), catalase A *CTA1* (ID: g07923, FC: −4.6), and catalase T *CTT1* (ID: g12987, FC:-8.4), and induced the homologous of thioredoxin peroxidase *TSA1* (ID: g11238, FC: 1.99). On the other hand, glutathione S-transferases (GST) are known for their ability to form GSH S-conjugates (GS-X) with xenobiotics compounds leading to their detoxification. In TF65-6, 17 of 18 differential GST were downregulated; among them was the homologous of *GTT2* (ID: g10968, FC: −7.18). Furthermore, the homologous of the major GS-X vacuolar transporter *YCF1* (ID: g01918, FC: −1.66) appeared repressed. In addition, we found that the expression of homologous of *GLO2* (ID: g03317, FC: −2.19) and *GLO4* (ID: g01227, FC: −2.35), which encode cytoplasmic and mitochondrial GLOII, respectively, were downregulated in TF65-6. Glyoxilase (GLO) system, which is a GSH-dependent pathway, carries out the detoxification of methylglyoxal, a by-product of glycolysis, which is a highly reactive endogenous glycation agent that can react with nucleic acids, lipids, and proteins.

##### 3.4.4.7. Signaling

In *Fusarium*, intracellular signaling pathways play critical roles in development, pathogenesis, and toxin biosynthesis (Yang et al., 2022). Different genes associated with these pathways were differentially expressed in *Fusarium* sp. TF65-6 (Supplementary Table S7). Homologous to the cAMP-dependent protein kinase catalytic subunit *TPK1* (ID: g07052, FC: 1.02), Calmodulin-dependent protein kinase *CMK2* (ID: g10716, FC: 2.19), and MAPKK involved in the control of cell wall integrity *MKK1* (ID: g06802, FC: 3.25) were induced, whereas homologous of the alpha catalytic subunit of casein kinase 2 (CK2) *CKA1* (ID: g10958, FC: −2.53) and MAPK involved in invasive growth *FUS3* (ID: g07562, FC: −1.6) were downregulated. In addition, several serine/threonine protein kinases, such as the homologous of *PKH2* (ID: g02283, FC: −2.82), which is involved in the sphingolipid-mediated signaling pathway (Dickson, 2008), and histidine kinases such as *nik1/os1* (ID: g04694, FC: 2.10), which are involved in osmotic stress responses and development (Alex et al., 1996; Schumacher et al., 1997; Motoyama et al., 2005) were differentially expressed in TF65-6 compared to WT, suggesting a disturbed performance of their associated pathways in the mutant. In addition, the homologous of the Gα subunit *fga3* (ID: g13557, FC: −2.66; Guo et al., 2016) and members of the Ras and Rho families of monomeric G proteins (IDs: g00831, g14938, g10730, g04952) were deregulated. Besides, we encountered two induced genes that encode phospholipase C (g00347 and g02979), which catalyzed the generation of two-second messenger molecules, inositol 1, 4, 5-triphosphate (IP3) and diacylglycerol (DAG) to activate de Ca^+2^ signaling through PKC.

##### 3.4.4.8. Regulation of gene expression

In this study, 79 genes related to the regulation of gene expression and belonging to different families were deregulated in *Fusarium* sp. TF65-6 (Supplementary Table S11). We found 24 induced DEGs and 55 repressed DEGs, enclosing putative TFs and transcriptional regulators. The most induced TF (ID: g01982, FC: 4.6) was a member of the fungal TFMHR superfamily (cl23766). Furthermore, DEGs homologous of *PacC* (ID: g00368, FC: 2.24) and *FlbC* (ID: g08719, FC: 1.41) were induced. The most repressed TF (ID: g08279, FC: −8.6) possesses domains that classify it in the fungal TF MHR (cl23766) and GAL4 (cl00068) superfamilies; interestingly, the homologous of *PDR1* (ID: g02781, FC: −2.2), considered a master regulator in multidrug resistance (Buechel and Pinkett, 2020), was also repressed. Diverse transcriptional regulators were differentially expressed in *Fusarium* sp. TF65-6 (Supplementary Table S11). The homologous of *TUP1/Rco-1*, the global repressor (ID: g04384, FC: 1.29; Yamashiro et al., 1996; Smith and Johnson, 2000), and *BYE1* (ID: g09018, FC: 1.29), a negative regulator of transcription elongation (Kinkelin et al., 2013; Pinskaya et al., 2014), were induced. Moreover, we identified induced genes involved in the chromatin remodeling and modification, as homologous of *NPL6* (ID: g14341, FC: 1.49) and *ARP9* (ID: g08340, FC: 1.05) components of the remodeling complexes RSC (Remodeling the Structure of Chromatin) and, RSC and SWI/SNF (switch/sucrose non-fermentable), respectively (Patel et al., 2019; Reyes et al., 2021). We observed, as induced, the homologous of *set-7* (ID: g03923, FC: 1.08), the catalytic PRC2 (Polycomb repressive complex 2) subunit in charge of establishing, maintaining, and recognizing the trimethylation of histone H3K27 (H3K27me3) (Jamieson et al., 2013), and *dim-2* (ID: g09568, FC: 1.98), which encodes a DNA methyltransferase (Kouzminova, 2001; He et al., 2020). Conversely, the homologous of *HST2* (ID: 01919, FC: −1.41), a sirtuin with histone deacetylase activity (Durand-Dubief et al., 2007), was repressed.

## 4. Discussion

Fungi modify gene expression during their life cycle and in response to environmental stimuli through the action of a variety of transcription factors (TFs). In fungal genomes, hundreds of TFs have been predicted and classified into approximately 80 TF families based on their DNA-binding domains. Interestingly, three TF families are fungal-specific: KilA-N/APSES, Mating-type MAT α1, and Copper fist (Shelest, 2017; Yu et al., 2023). However, few members of these families have been characterized. In phytopathogenic fungi, members of 10 families have been studied; specifically in *F. oxysporum*, it was recently reported that from nearly 800 TFs predicted, only 26 TFs had been functionally characterized (John et al., 2021; Zuriegat et al., 2021), highlighting the universe of TFs that remains to be characterized. The KilA-N/APSES family is described as exclusive in fungi; however, this domain is found in DNA viruses of prokaryotes and eukaryotes (Iyer et al., 2002). Some TFs of the KilA-N family have a solid connection to cell cycle regulation but also diversified their function in other aspects of fungal cell biology (Zhao et al., 2015; Medina et al., 2019). In this study, we identified five KilA-N TFs in *Fusarium* sp. INECOL_BM-06 associated with *Xylosandrus morigerus* (Figure 1A; Supplementary Table S3) in accordance with the distribution and conservation encountered in fungal species of the subphylum Pezizomycotina (Medina et al., 2019). The Bqt4 is the youngest and least distributed member of the KilA-N family and, therefore, less studied. Here, FspTF, a homologous of Bqt4 of *S. pombe* (Chikashige et al., 2009), and MtgA of *A. nidulans* (Chemudupati et al., 2016) were functionally characterized in *Fusarium* sp. associated with *X. morigerus*. Bqt4 and MtgA are proteins embedded in the inner nuclear membrane (INM), and the KilA-N domain in Bqt4 of *S. pombe* is reported to have moderate and unspecific DNA-binding activity and to mediate protein–protein interactions (Chikashige et al., 2009, 2010; Hu et al., 2019a,b). In this study, we generated an edited strain, TF65-6, by the CRISPR/Cas9 system, in which Fsp*tf* was downregulated (Supplementary Figure S3) and the protein was predicted to lack the KilA-N domain (Figure 1E) and, in consequence, it was suggested to fail in both binding DNA and protein interaction through this domain.

The phylogenetic analysis indicated that FspTF is less related to Bqt4 and MtgA, suggesting that functional differences exist between them; even Bqt4 and MtgA were not highly related, denoting their differences regarding telomere tethering, among others. Instead, FspTF formed a cluster with the orthologous of *Fusarium* species that were more related to the orthologous of *N. crassa*, which have not been characterized, meaning that this is the first study exploring this TF in *Fusarium* spp.

The Fsp*tf* expression analysis that we conducted in different conditions (Figure 1C) indicated a possible role in different biological processes of *Fusarium* sp., including growth and pathogenesis. In congruence, the edition of Fsp*tf* in *Fusarium* sp. resulted in various phenotypical changes, such as increased mycelial growth, reduction of conidia production (but accelerated conidia germination rate), decreased virulence, and secondary metabolites production, and it also modified the response to diverse stresses (Figures 2–4). In addition, Δ*tf*, a replacement mutant, showed the same phenotypical changes as TF65-6, regarding mycelial growth, conidia germination rate, virulence, secondary metabolites production, and responses to certain stresses (Supplementary Figure S6–S8). The high-throughput data provided evidence of gene candidates that may be regulated by the action of FspTF to modulate pigment production, growth, and virulence.

### 4.1. FspTF control pigment production

TF65-6 showed a lack of mycelium and medium pigmentation (Figure 2A), down-accumulation of the pigments javanicin and fusarubin (Table 1), and those genes belonging to the FSR cluster were repressed (Table 2). Altogether, these results can explain the unpigmented phenotype and give clues about the possible direct or indirect targets of FspTF. In addition, we informed for the first time that *Fusarium* sp. INECOL_BM-06 has the capacity to produce javanicin and fusarubin, which is relevant given the ecological niche of this fungus and that these pigments are reported with antifungal and antibacterial activity (Li et al., 2020) and, in the ambrosia *F. ambrosium*, are suggested to prevent the invasion of endophytic fungi in galleries of *Euwallacea formicatus*, protecting its habitat (Kehelpannala et al., 2018). This is in agreement with the succession of metabolic roles that occur between bacteria and fungi that inhabit the galleries to guarantee the optimal development of ambrosia beetles (Ibarra-Juarez et al., 2020).

### 4.2. Growth is controlled by the expression of transmembranal nutrient transporters, regulated by FspTF

In TF65-6, most DEG-encoding transporters were induced (Supplementary Table S8), denoting the active income of nutrients that contribute to the increased colony growth. Fungi use a variety of sugars such as hexoses as primary carbon source; in yeast, the transport of hexoses is mediated by hexose transporters (Hxt, Hxt1 to Hxt17) (Barata-Antunes et al., 2021). TF65-6 overexpresses homologous of *HXT1*, *HXT9*, *HXT13*, *HXT15*, and *HXT17*. HXT1 is a low-affinity glucose transporter (Bisson et al., 2016), HXT9 is a functional glucose carrier (Wieczorke et al., 1999), and HXT13, HXT15, and HXT17 are polyol transporters (Jordan et al., 2016); the expression of *HXT13* and *HXT15* was induced in the presence of non-fermentable carbon sources (Greatrix and van Vuuren, 2006). However, the homologous of *CAT8*, a TF required for the growth in non-fermentative conditions (Hedges et al., 1995; Haurie et al., 2001), was repressed. Furthermore, the homologous of *HXT2* and *HXT6*, high-affinity transporters, were repressed. The same situation was observed with regard to the uptake of maltose, with genes encoding maltose permeases being induced; conversely, the TFs that regulate maltose utilization, *MAL13* and *MAL33* (Orikasa et al., 2018), were repressed. Taken together, these results suggest that sufficiency of glucose is sensed by the mutant but can incorporate alternative C sources, although it cannot use them efficiently. This hypothesis must be proved in future research work.

On the other hand, TF65-6 repressed specific genes encoding TFs related to the biosynthesis of certain amino acids, *ARG81*, *Lys14*, and *ARO80*, and overexpressed transporters for different amino acids (Supplementary Table S8), denoting the necessity of taking them from the environment, which, in addition, can be used as carbon and nitrogen sources and as substrates for the biosynthesis of other biomolecules, such as NAD^+^, folate, glutathione, nucleotides, polyamines, and phospholipids (Bianchi et al., 2019). In addition, the repression of *nit-4* (Yuan et al., 1991; Huberman et al., 2021), the induction of *UGA3* (Cardillo et al., 2011; Sylvain et al., 2011), and the induction of transporters of non-preferred nitrogen sources as urea, purines, oligopeptides, and allantoate (Supplementary Table S8) indicates the possibility of assimilating alternative nitrogen sources. In this context, TF65-6 seems to respond to its intracellular state by regulating the transport systems across biological membranes to uptake essential nutrients to sustain active growth, a common compensation mechanism in fungi (Ramos et al., 2016).

### 4.3. FspTF is relevant for controlling the pathogenic process

Our results suggest that TF65-6 does not entirely lose its pathogenicity but dramatically changes the progression velocity of the infection process. The hypothesis above is sustained by the characterization of several pathosystems using histochemical tests and analyzing some plant defense marker genes (Figures 4–5). However, the changes depend totally on the host tested (Supplementary Figure S4). Based on these observations, we encountered genes that can be related to the phenotype in pathogenesis. In this sense, our data suggest that TF65-6 can transgress the cuticle and degrade the plant cell wall in order to establish within the host, denoted by the induced genes related to lipid degradation, hydrolytic enzymes, and oxidases (Supplementary Table S9) and the evident establishment of the infection (Figure 4; Supplementary Figure S4). Nevertheless, inside the host, TF65-6 cannot take nutrients to support the progression of the infection, given that most of the proteases differentially expressed were repressed (Supplementary Table S9).

On the other hand, the genes related to effectors were induced (Table 2). Therefore they could have a minor role in pathogenesis, could participate in the establishment of the infection, but not in its progression, and could allow the induction of defense responses in *P. nigra* (Figure 5; Zeng et al., 2023). This is consistent with previous studies in which it was proven that phytopathogenic species of *Fusarium* genus associated with ambrosia beetles strongly induced genes related to defense in their host (Pérez-Torres et al., 2021). In addition, TF65-6 downregulated different PKS, including that involved in the synthesis of fusaric acid, recognized as a virulence factor in *Fusarium* (López-Díaz et al., 2018), and diverse NRPS (Table 2), which can participate in the synthesis of biologically active cyclic depsipeptides and other phytotoxic nitrogen-containing metabolites, which are important players for the development of plant disease symptoms (Xu et al., 2021). Moreover, fumonisins are reported as potential virulence factors, with FB1 being the most relevant in this regard (Sánchez-Rangel et al., 2012; Xie et al., 2021). TF65-6 down-accumulated fumonisin B4 (Table 1) and repressed *FUM13* (Table 2), which can contribute to the observed phenotype related to virulence. Fusaproliferin was over-accumulated, and genes of the *FUS* cluster involved in the biosynthesis of fusarins were downregulated in TF65-6. The role of these mycotoxins in the ecology of *Fusarium* is not clear, and their function as virulence factors, in development, in protection against competitors and predators, and as an adaptation mechanism cannot be discarded (Geisen et al., 2017). Additionally, in *F. graminearum*, ABC-transporters have a role in virulence and azole tolerance, specifically, FgABC3, suggesting encoding a transporter protecting the fungus from host-derived antifungal molecules (Abou Ammar et al., 2013). TF65-6 downregulated genes encoding for ABC transporters (Supplementary Table S8) and the edited strain had enhanced sensitivity to azole fungicides (Figures 8A,B). Thus, these genes could also have an active role in virulence in *Fusarium* sp. However, we cannot discard a specific transcriptional profile during the interaction with the host, which makes it possible to identify genes more closely related to the infection process.

### 4.4. Conidiation and conidia germination were driven by FspTF

In fungi, the production of conidiophores requires the sequential activity of three proteins, BrlA, AbaA, and WetA, and five upstream regulators, FluG, FlbB, FlbC, FlbD, and FlbE (Park and Yu, 2012; Baltussen et al., 2018). In *Fusarium*, some of these regulatory proteins have been characterized (Ajmal et al., 2022). In TF65-6, the TF FlbC was described as induced (Supplementary Table S11), which plays a key role in asexual development in *F. verticilloides* (Malapi-Wight et al., 2014). Our data showed that homologous *fluG* (ID: g09766, FC: −1.52) and *wetA* (ID: g09928, FC: −1.27) were repressed; in *F. graminearum*, WetA is required for conidiogenesis and conidium maturation (Son et al., 2014). Together, these data suggest that in TF65-6, the genetic pathway for spore formation is deregulated, leading to low production of conidia. However, TF65-6 showed an accelerated germination rate, suggesting an enhanced transcriptional activity in its conidia. Given the sampling time of our transcriptional data, we were able to correlate DEGs with the last morphotype of the conidia germination (Baltussen et al., 2018; Verburg et al., 2022), polarized growth. During polarized growth, genes that modulate cellular growth, metabolism, and DNA replication were upregulated predominantly in conjunction with cell wall biosynthesis- and remodeling- genes (Baltussen et al., 2018; Riquelme et al., 2018; Verburg et al., 2022). In TF65-6, there were DEGs involved in hyphal growth; among these were chitin synthases (ID: g00964 and g14280: induced; g01829, g02154, g00086, and g07954: repressed), chitinases (ID: g06501, g11168, and g12474: induced; g09277, g09278, g02824, g07091, g03775, and g09276: repressed), septins (g03367, g08731, and g07121: induced), and cytoskeleton proteins (g05508, g0332, g14985, g08857, g13820, g14562, and g14828: induced; g03678: repressed; Supplementary Table S7). Furthermore, genes involved in the cell cycle were differential; the homologous of the master regulator Cdk *CDC28* (ID: g09383, FC: −1.81) was downregulated; interestingly, two homologous of cyclins, *CLB2* (ID: g14263, FC: 1.31) and *CLB4* (ID: g03342, FC: 1.40), which promote the transition from G2 to M phase, were induced. In addition, homologous of *SWE1* (ID: g13596, FC: 1.88), a kinase that phosphorylates CDC28 and inhibits entry into M phase, and *MIH1* (ID: g14646, FC: 1.9), the phosphatase that modifies CDC28 for entry into M phase, were also induced (Dörter and Momany, 2016), suggesting that the stimulation to entry to M phase of the cell cycle was enhanced. Combining these data suggests that conidia germination was favored, ending in the formation of the germ tube, which continued to grow, taking place of the vegetative growth, and allowing the formation of a bigger colony in the mutant.

### 4.5. Stress responses coordinated by FspTF

Coordinated stress responses are essential for fungi to survive in hostile conditions encountered in the environment. In the mutant, most of the key enzymes of metabolic systems that remove cell-damaging ROS, such as superoxide dismutase, catalase, and glutathione/glutaredoxin/thioredoxin (Pócsi et al., 2004), were downregulated (Supplementary Table S10), which explains its sensibility to oxidative stress. Besides, osmotic stress is known to generate intracellular oxidative stress, and the deregulation of ROS scavenging pathways can participate in the low tolerance to this stress (Figure 3).

On the other hand, the less sensitive phenotype of the edited strain during HU treatment was entirely unexpected. However, our subsequent analysis identified some candidate DEGs that may promote this phenotype. HU is an inhibitor of the ribonucleotide reductase (RNR) that catalyzes the synthesis of deoxyribonucleotides from ribonucleotides, slowing replication forks and arresting the cell cycle in the S phase; in consequence, the DNA replication checkpoint (DRC) pathway is activated to upregulate RNR and increase dNTPs production, which promotes fork progression (Xu et al., 2016). In TF65-6, some components of the DRC were induced, such as the homologous of *MEC1* (ID: g07221, FC: 1.13) and *CHK1* (ID: g14022, FC: 1.08) (Dörter and Momany, 2016). Furthermore, *RNR1* (ID: g00986, FC: 1.02), homologous of the large subunit of the RNR, was induced. These genes can contribute to the observed tolerance of TF65-6 to HU. In addition, there is evidence that the ergosterol pathway is essential to counter this stress since the mutant of *S. pombe* in *erg11*, with sterol deficiency, is more sensitive to HU treatment (Xu et al., 2016). Our data suggest that ergosterol biosynthesis was altered in the TF65-6 edited strain since *ERG* genes were differentially expressed (Table 2; Supplementary Figure S5), and mainly *ERG11* was upregulated, which can improve the tolerance to this genotoxic. Finally, HU also rescues the conditional lethality of mutants in *S. cerevisiae*, defective in diverse cellular pathways, including chromosome segregation, redox homeostasis, and sterol biosynthesis by an unknown mechanism (McCulley et al., 2014). Given the alterations of diverse cellular pathways in TF65-6, a chemical suppression exerted by HU can function in TF65-6, evidenced by the alleviated effect of the compound during growth.

### 4.6. Signal transduction and gene expression regulated by FspTF

In fungi, intra- and extracellular stimuli are translated into specific physiologic responses, through transcription regulation, by the precise coordination of signaling pathways that are reported to play an essential role in growth, development, pathogenesis, and mycotoxin biosynthesis (Guo et al., 2016; Barman et al., 2018; Yang et al., 2022). In TF65-6, we encountered DEGs that encoded components of G protein, MAPK, PKA, and PKC signaling pathways. Reversible protein phosphorylation by protein kinases (PK) regulates various fungal biological processes. In TF65-6, different PKs are differentially expressed, and some, such as *CKA1*, are important for various aspects of growth, developmental and infection processes in *F. graminearum* (Wang et al., 2011). Small Ras GTPases, monomeric G proteins, also participate in signal transduction as molecular switches with multiple functions; in TF65-6, *RAS2* and *RHO1* were induced, which are reported with a role in gene expression regulation, polar growth, membrane traffic, and morphogenesis in fungi (Dautt-Castro et al., 2021). Furthermore, we find specific TFs involved in the regulation of metabolic pathways (Supplementary Table S8) and other diverse TFs belonging to different families and chromatin remodelers (Supplementary Table S11). Thus, given that in the edited strain, multiple components of signaling pathways were deregulated, and the signaling pathways were interconnected and functionally pleiotropic, it was most likely that the phenotypes that we identified related to growth, pathogenesis, and stress were also the consequence of the “new” complex network which was functional in the edited strain.

In summary, this study revealed that FspTF plays a relevant role in overall growth, development, metabolism, stress responses, and pathogenesis by regulating gene expression. However, the open question that arises is which is/are the mechanism(s) and which is/are the direct target(s) of FspTF in *Fusarium* sp. associated with *X. morigerus*. Recently, it was encountered that Bqt4 interacts directly or indirectly with proteins with diverse cellular functions such as nucleoplasmic transport, protein degradation by 19S proteasome, cytoplasmic translation, cell cycle, and lipid metabolism, among others (Hirano et al., 2023a,b). It was proposed that Bqt4 interacts with proteins related to histone modification, ubiquitination, and DNA repair (Hu et al., 2019a), and participates actively with the function of Lem2, an INM protein (Ebrahimi et al., 2018; Hirano et al., 2018) with roles in an array of genome-regulatory functions including epigenetic silencing, DNA replication, and post-transcriptional regulation (Banday et al., 2016; Barrales et al., 2016; Tange et al., 2016; Ebrahimi et al., 2018; Hirano et al., 2018; Martín Caballero et al., 2022). Protein–protein interaction in the nuclear envelope can be the main molecular mechanism involved in how FspTF can function as a major regulator in *Fusarium* sp; however, this hypothesis remains to be investigated in future work.

## 5. Conclusion

Despite transcription factors being key players in the regulation of biological processes in all organisms, a universe of fungal transcription factors remains uncharacterized, highlighting many aspects of fungal biology expected to be studied. In this work, we identified that FspTF, a member of the fungal-specific family of transcription factor KilA-N/APSES, of *Fusarium* sp. associated with the ambrosia beetle *Xylosandrus morigerus*, is involved in growth, conidia production and germination, responses to stress, and pathogenesis, revealing new aspects of the TFs grouped in the KilA-N/APSES family in *Fusarium* genus, with particular emphasis in those species that are symbiotes of ambrosia beetles. These findings open the possibility of identifying targets for the prevention and mitigation of plant diseases caused by the ambrosia complex.

## Data availability statement

The datasets presented in this study can be found in online repositories. The names of the repository/repositories and accession number(s) can be found in the article/Supplementary material.

## Author contributions

NC-V and DS-R: contributed to the conceptual design of the study, formal analysis, and wrote the manuscript. NC-V, LM-R, EI-L, JM-V, BR-H, and JG-A: methodology. EI-L: transcriptome assembly, annotation, and statistical analysis. JM-V and JG-A: untargeted metabolomics analysis. DS-R funding acquisition and project administration. All authors read and approved the final manuscript.

## Funding

This work was supported by Consejo Nacional de Humanidades, Ciencias y Tecnologías (CONAHCYT) through the Fondo Institucional de Fomento Regional para el Desarrollo Científico, Tecnológico y de Innovación (Fordecyt) Grant 292399 (currently named Programa Presupuestario F003) and by INECOL A.C. (20047/90001).

## Conflict of interest

The authors declare that the research was conducted in the absence of any commercial or financial relationships that could be construed as a potential conflict of interest.

## Publisher’s note

All claims expressed in this article are solely those of the authors and do not necessarily represent those of their affiliated organizations, or those of the publisher, the editors and the reviewers. Any product that may be evaluated in this article, or claim that may be made by its manufacturer, is not guaranteed or endorsed by the publisher.

## Footnotes

^1^ http://smart.embl-heidelberg.de/

^2^ http://www.ncbi.nlm.nih.gov/cdd/

^3^ https://www.ebi.ac.uk/interpro/

^4^ http://www.softberry.com/berry.phtml?topic=fgenesh&group=programs&subgroup=gfind

^5^ https://blast.ncbi.nlm.nih.gov/Blast.cgi

^6^ https://mycocosm.jgi.doe.gov/Necha2/Necha2.home.html

^7^ https://services.healthtech.dtu.dk/service.php?SignalP

^8^ https://services.healthtech.dtu.dk/service.php?DeepLoc

^9^ https://effectorp.csiro.au/

^10^ https://www.ncbi.nlm.nih.gov/Structure/bwrpsb/bwrpsb.cgi

^11^ http://crispor.tefor.net/

^12^ https://www.metaboanalyst.ca/MetaboAnalyst/home.xhtml

^13^ https://www.ncbi.nlm.nih.gov/

^14^ http://biit.cs.ut.ee/gprofiler/

## References

[ref1] Abou Ammar G. Tryono R. Döll K. Karlovsky P. Deising H. B. Wirsel S. G. R. (2013). Identification of ABC transporter genes of *Fusarium graminearum* with roles in azole tolerance and/or virulence. PLoS One 8:e79042. doi: 10.1371/journal.pone.0079042, PMID: 24244413 PMC3823976

[ref2] Aguilera J. Randez-Gil F. Prieto J. A. (2007). Cold response in *Saccharomyces cerevisiae*: new functions for old mechanisms. FEMS Microbiol. Rev. 31, 327–341. doi: 10.1111/j.1574-6976.2007.00066.x, PMID: 17298585

[ref3] Ajmal M. Hussain A. Ali A. Chen H. Lin H. (2022). Strategies for controlling the sporulation in *Fusarium* spp. J Fungi (Basel) 9:10. doi: 10.3390/jof9010010, PMID: 36675831 PMC9861637

[ref4] Alex L. A. Borkovich K. A. Simon M. I. (1996). Hyphal development in neurospora crassa: involvement of a two-component histidine kinase. Proc. Natl. Acad. Sci. U. S. A. 93, 3416–3421. doi: 10.1073/pnas.93.8.3416, PMID: 8622950 PMC39623

[ref5] Amar N. Messenguy F. El Bakkoury M. Dubois E. (2000). ArgRII, a component of the ArgR-Mcm1 complex involved in the control of arginine metabolism in *Saccharomyces cerevisiae*, is the sensor of arginine. Mol. Cell. Biol. 20, 2087–2097. doi: 10.1128/MCB.20.6.2087-2097.2000, PMID: 10688655 PMC110825

[ref6] Andersen H. F. Jordal B. H. Kambestad M. Kirkendall L. R. (2012). Improbable but true: the invasive inbreeding ambrosia beetle *Xylosandrus morigerus* has generalist genotypes: general-purpose genotypes in an invasive species. Ecol. Evol. 2, 247–257. doi: 10.1002/ece3.58, PMID: 22408740 PMC3297192

[ref7] Awuchi C. G. Ondari E. N. Ogbonna C. U. Upadhyay A. K. Baran K. Okpala C. O. R.. (2021). Mycotoxins affecting animals, foods, humans, and plants: types, occurrence, toxicities, action mechanisms, prevention, and detoxification strategies-a revisit. Foods 10:1279. doi: 10.3390/foods10061279, PMID: 34205122 PMC8228748

[ref8] Baltussen T. J. H. Coolen J. P. M. Zoll J. Verweij P. E. Melchers W. J. G. (2018). Gene co-expression analysis identifies gene clusters associated with isotropic and polarized growth in *Aspergillus fumigatus* conidia. Fungal Genet. Biol. 116, 62–72. doi: 10.1016/j.fgb.2018.04.013, PMID: 29705402

[ref9] Banday S. Farooq Z. Rashid R. Abdullah E. Altaf M. (2016). Role of inner nuclear membrane protein complex Lem2-Nur1 in heterochromatic gene silencing. J. Biol. Chem. 291, 20021–20029. doi: 10.1074/jbc.M116.743211, PMID: 27451393 PMC5025688

[ref10] Barata-Antunes C. Alves R. Talaia G. Casal M. Gerós H. Mans R.. (2021). Endocytosis of nutrient transporters in fungi: the ART of connecting signaling and trafficking. Comput. Struct. Biotechnol. J. 19, 1713–1737. doi: 10.1016/j.csbj.2021.03.013, PMID: 33897977 PMC8050425

[ref11] Barman A. Gohain D. Bora U. Tamuli R. (2018). Phospholipases play multiple cellular roles including growth, stress tolerance, sexual development, and virulence in fungi. Microbiol. Res. 209, 55–69. doi: 10.1016/j.micres.2017.12.012, PMID: 29580622

[ref12] Barrales R. R. Forn M. Georgescu P. R. Sarkadi Z. Braun S. (2016). Control of heterochromatin localization and silencing by the nuclear membrane protein Lem2. Genes Dev. 30, 133–148. doi: 10.1101/gad.271288.115, PMID: 26744419 PMC4719305

[ref13] Bateman C. Šigut M. Skelton J. Smith K. E. Hulcr J. (2016). Fungal associates of the *Xylosandrus compactus* (Coleoptera: Curculionidae, Scolytinae) are spatially segregated on the insect body. Environ. Entomol. 45, 883–890. doi: 10.1093/ee/nvw070, PMID: 27357160

[ref14] Bianchi F. Van’t Klooster J. S. Ruiz S. J. Poolman B. (2019). Regulation of amino acid transport in *Saccharomyces cerevisiae*. Microbiol. Mol. Biol. Rev. 83, e00024–e00019. doi: 10.1128/MMBR.00024-19 31619504 PMC7405077

[ref15] Bisson L. F. Fan Q. Walker G. A. (2016). Sugar and glycerol transport in *Saccharomyces cerevisiae*. Adv. Exp. Med. Biol. 892, 125–168. doi: 10.1007/978-3-319-25304-6_6 26721273

[ref16] Bolger A. M. Lohse M. Usadel B. (2014). Trimmomatic: a flexible trimmer for Illumina sequence data. Bioinformatics 30, 2114–2120. doi: 10.1093/bioinformatics/btu170, PMID: 24695404 PMC4103590

[ref17] Brown D. W. Butchko R. A. E. Busman M. Proctor R. H. (2012). Identification of gene clusters associated with fusaric acid, fusarin, and perithecial pigment production in *Fusarium verticillioides*. Fungal Genet. Biol. 49, 521–532. doi: 10.1016/j.fgb.2012.05.010, PMID: 22652150

[ref18] Buechel E. R. Pinkett H. W. (2020). Transcription factors and ABC transporters: from pleiotropic drug resistance to cellular signaling in yeast. FEBS Lett. 594, 3943–3964. doi: 10.1002/1873-3468.13964, PMID: 33089887

[ref19] Butchko R. A. E. Plattner R. D. Proctor R. H. (2003). *FUM13* encodes a Short chain dehydrogenase/reductase required for C-3 carbonyl reduction during Fumonisin biosynthesis in *Gibberella moniliformis*. J. Agric. Food Chem. 51, 3000–3006. doi: 10.1021/jf0262007, PMID: 12720383

[ref20] Cappellini R. A. Peterson J. L. (1965). Macroconidium formation in submerged cultures by a nonsporulating strain of Gibberella Zeae. Mycologia 57, 962–966. doi: 10.1080/00275514.1965.12018285

[ref21] Cardillo S. B. Correa García S. Bermúdez Moretti M. (2011). Common features and differences in the expression of the three genes forming the UGA regulon in *Saccharomyces cerevisiae*. Biochem. Biophys. Res. Commun. 410, 885–889. doi: 10.1016/j.bbrc.2011.06.086, PMID: 21708130

[ref22] Carreras-Villaseñor N. Rodríguez-Haas J. B. Martínez-Rodríguez L. A. Pérez-Lira A. J. Ibarra-Laclette E. Villafán E.. (2022). Characterization of two *Fusarium solani* species complex isolates from the Ambrosia beetle *Xylosandrus morigerus*. JoF 8:231. doi: 10.3390/jof8030231, PMID: 35330233 PMC8956061

[ref23] Carrillo D. Cruz L. Kendra P. Narvaez T. Montgomery W. Monterroso A.. (2016). Distribution, pest status and fungal associates of *Euwallacea* nr. *fornicatus* in Florida avocado groves. Insects 7:55. doi: 10.3390/insects7040055, PMID: 27754408 PMC5198203

[ref24] Carrillo J. D. Dodge C. Stouthamer R. Eskalen A. (2020). Fungal symbionts of the polyphagous and Kuroshio shot hole borers (*Coleoptera*: *Scolytinae*, *Euwallacea* spp.) in California can support both ambrosia beetle systems on artificial media. Symbiosis 80, 155–168. doi: 10.1007/s13199-019-00652-0

[ref25] Ćeranić A. Svoboda T. Berthiller F. Sulyok M. Samson J. M. Güldener U.. (2021). Identification and functional characterization of the gene cluster responsible for Fusaproliferin biosynthesis in *Fusarium proliferatum*. Toxins (Basel) 13:468. doi: 10.3390/toxins13070468, PMID: 34357940 PMC8310001

[ref26] Charron M. J. Dubin R. A. Michels C. A. (1986). Structural and functional analysis of the MALI locus of *Saccharomyces cerevisiae*. Mol. Cell. Biol. 6, 3891–3899. doi: 10.1128/mcb.6.11.3891-3899.1986, PMID: 3025617 PMC367152

[ref27] Chemudupati M. Osmani A. H. Osmani S. A. (2016). A mitotic nuclear envelope tether for Gle1 also affects nuclear and nucleolar architecture. MBoC 27, 3757–3770. doi: 10.1091/mbc.e16-07-0544, PMID: 27630260 PMC5170558

[ref28] Chikashige Y. Haraguchi T. Hiraoka Y. (2010). Nuclear envelope attachment is not necessary for telomere function in fission yeast. Nucleus 1, 481–486. doi: 10.4161/nucl.1.6.13113, PMID: 21327090 PMC3027050

[ref29] Chikashige Y. Yamane M. Okamasa K. Tsutsumi C. Kojidani T. Sato M.. (2009). Membrane proteins Bqt3 and −4 anchor telomeres to the nuclear envelope to ensure chromosomal bouquet formation. J. Cell Biol. 187, 413–427. doi: 10.1083/jcb.200902122, PMID: 19948484 PMC2779253

[ref30] Choudhury B. I. Whiteway M. (2018). Evolutionary transition of GAL regulatory circuit from generalist to specialist function in Ascomycetes. Trends Microbiol. 26, 692–702. doi: 10.1016/j.tim.2017.12.008, PMID: 29395731

[ref31] Concordet J.-P. Haeussler M. (2018). CRISPOR: intuitive guide selection for CRISPR/Cas9 genome editing experiments and screens. Nucleic Acids Res. 46, W242–W245. doi: 10.1093/nar/gky354, PMID: 29762716 PMC6030908

[ref32] Connolly L. R. Smith K. M. Freitag M. (2013). The *Fusarium graminearum* histone H3 K27 methyltransferase KMT6 regulates development and expression of secondary metabolite gene clusters. PLoS Genet. 9:e1003916. doi: 10.1371/journal.pgen.1003916, PMID: 24204317 PMC3814326

[ref33] Daudi A. O’Brien J. (2012). Detection of hydrogen peroxide by DAB staining in Arabidopsis leaves. Bioprotocol 2:e263. doi: 10.21769/BioProtoc.263, PMID: 27390754 PMC4932902

[ref34] Dautt-Castro M. Rosendo-Vargas M. Casas-Flores S. (2021). The small GTPases in fungal Signaling conservation and function. Cells 10:1039. doi: 10.3390/cells10051039, PMID: 33924947 PMC8146680

[ref35] Dickson R. C. (2008). Thematic review series: sphingolipids. New insights into sphingolipid metabolism and function in budding yeast. J. Lipid Res. 49, 909–921. doi: 10.1194/jlr.R800003-JLR200, PMID: 18296751 PMC2311445

[ref36] Dörter I. Momany M. (2016). Fungal cell cycle: a unicellular versus multicellular comparison. Microbiol Spectr 4:4.6.28. doi: 10.1128/microbiolspec.FUNK-0025-2016 28087934

[ref37] Dubois E. Messenguy F. (1985). Isolation and characterization of the yeast ARGRII gene involved in regulating both anabolism and catabolism of arginine. Mol. Gen. Genet. 198, 283–289. doi: 10.1007/BF00383008, PMID: 3884975

[ref38] Durand-Dubief M. Sinha I. Fagerström-Billai F. Bonilla C. Wright A. Grunstein M.. (2007). Specific functions for the fission yeast Sirtuins Hst2 and Hst4 in gene regulation and retrotransposon silencing. EMBO J. 26, 2477–2488. doi: 10.1038/sj.emboj.7601690, PMID: 17446861 PMC1868902

[ref39] Dzurenko M. Hulcr J. (2022). Ambrosia beetles. Curr. Biol. 32, R61–R62. doi: 10.1016/j.cub.2021.11.043, PMID: 35077686

[ref40] Ebrahimi H. Masuda H. Jain D. Cooper J. P. (2018). Distinct ‘safe zones’ at the nuclear envelope ensure robust replication of heterochromatic chromosome regions. Elife 7:e32911. doi: 10.7554/eLife.32911, PMID: 29722648 PMC5933923

[ref41] Ekwomadu T. I. Akinola S. A. Mwanza M. (2021). *Fusarium* mycotoxins, their metabolites (free, emerging, and masked), food safety concerns, and health impacts. Int. J. Environ. Res. Public Health 18:11741. doi: 10.3390/ijerph182211741, PMID: 34831498 PMC8618243

[ref42] Eskalen A. Gonzalez A. Wang D. H. Twizeyimana M. Mayorquin J. S. Lynch S. C. (2012). First report of a *Fusarium* sp. and its vector tea shot hole borer (*Euwallacea fornicatus*) causing *Fusarium* dieback on avocado in California. Plant Dis. 96:1070. doi: 10.1094/PDIS-03-12-0276-PDN, PMID: 30727226

[ref43] Fabri J. H. T. De Sá N. P. Malavazi I. Del Poeta M. (2020). The dynamics and role of sphingolipids in eukaryotic organisms upon thermal adaptation. Prog. Lipid Res. 80:101063. doi: 10.1016/j.plipres.2020.101063, PMID: 32888959 PMC7674228

[ref44] Feller A. Dubois E. Ramos F. And Pieirard A. (1994). Repression of the genes for lysine biosynthesis in *Saccharomyces cerevisiae* is caused by limitation of Lysl4-dependent transcriptional activation. Mol. Cell. Biol. 14, 6411–6418. doi: 10.1128/mcb.14.10.6411-6418.1994, PMID: 7935367 PMC359171

[ref45] Fernández-Bautista N. Domínguez-Núñez J. Moreno M. M. Berrocal-Lobo M. (2016). Plant tissue trypan blue staining during phytopathogen infection. Bioprotocol 6:2078. doi: 10.21769/BioProtoc.2078

[ref46] Freeman S. Sharon M. Maymon M. Mendel Z. Protasov A. Aoki T.. (2013). *Fusarium euwallaceae* sp. nov.–a symbiotic fungus of *Euwallacea* sp., an invasive ambrosia beetle in Israel and California. Mycologia 105, 1595–1606. doi: 10.3852/13-066, PMID: 23928415

[ref47] Geisen R. Touhami N. Schmidt-Heydt M. (2017). Mycotoxins as adaptation factors to food related environments. Curr. Opin. Food Sci. 17, 1–8. doi: 10.1016/j.cofs.2017.07.006, PMID: 33003323

[ref48] Gouy M. Guindon S. Gascuel O. (2010). SeaView version 4: a multiplatform graphical user Interface for sequence alignment and phylogenetic tree building. Mol. Biol. Evol. 27, 221–224. doi: 10.1093/molbev/msp259, PMID: 19854763

[ref49] Greatrix B. W. van Vuuren H. J. J. (2006). Expression of the HXT13, HXT15 and HXT17 genes in Saccharomyces cerevisiae and stabilization of the HXT1 gene transcript by sugar-induced osmotic stress. Curr. Genet. 49, 205–217. doi: 10.1007/s00294-005-0046-x, PMID: 16397765

[ref50] Grosman D. M. Eskalen A. Brownie C. (2019). Evaluation of Emamectin benzoate and propiconazole for Management of a new Invasive Shot Hole Borer (*Euwallacea* nr. *fornicatus*, Coleoptera: Curculionidae) and symbiotic Fungi in California sycamores. J. Econ. Entomol. 112, 1267–1273. doi: 10.1093/jee/toy423, PMID: 30649416 PMC6529917

[ref51] Gruber-Dorninger C. Novak B. Nagl V. Berthiller F. (2017). Emerging mycotoxins: beyond traditionally determined food contaminants. J. Agric. Food Chem. 65, 7052–7070. doi: 10.1021/acs.jafc.6b03413, PMID: 27599910

[ref52] Guevara-Avendaño E. Bejarano-Bolívar A. A. Kiel-Martínez A.-L. Ramírez-Vázquez M. Méndez-Bravo A. Von Wobeser E. A.. (2019). Avocado rhizobacteria emit volatile organic compounds with antifungal activity against *Fusarium solani*, *Fusarium* sp. associated with Kuroshio shot hole borer, and Colletotrichum gloeosporioides. Microbiol. Res. 219, 74–83. doi: 10.1016/j.micres.2018.11.009, PMID: 30642469

[ref53] Guevara-Avendaño E. Bravo-Castillo K. R. Monribot-Villanueva J. L. Kiel-Martínez A. L. Ramírez-Vázquez M. Guerrero-Analco J. A.. (2020). Diffusible and volatile organic compounds produced by avocado rhizobacteria exhibit antifungal effects against *Fusarium kuroshium*. Braz. J. Microbiol. 51, 861–873. doi: 10.1007/s42770-020-00249-6, PMID: 32166656 PMC7455650

[ref54] Guevara-Avendaño E. Carrillo J. D. Ndinga-Muniania C. Moreno K. Méndez-Bravo A. Guerrero-Analco J. A.. (2018). Antifungal activity of avocado rhizobacteria against *Fusarium euwallaceae* and *Graphium* spp., associated with *Euwallacea* spp. nr. *fornicatus*, and *Phytophthora cinnamomi*. Antonie Van Leeuwenhoek 111, 563–572. doi: 10.1007/s10482-017-0977-5, PMID: 29124466

[ref55] Gugliuzzo A. Biedermann P. H. W. Carrillo D. Castrillo L. A. Egonyu J. P. Gallego D.. (2021). Recent advances toward the sustainable management of invasive *Xylosandrus* ambrosia beetles. J. Pest. Sci. 94, 615–637. doi: 10.1007/s10340-021-01382-3

[ref56] Guo L. Yang Y. Yang L. Wang F. Wang G. Huang J. (2016). Functional analysis of the G-protein α subunits FGA1 and FGA3 in the banana pathogen *Fusarium oxysporum* f. sp. *cubense*. Physiol. Mol. Plant Pathol. 94, 75–82. doi: 10.1016/j.pmpp.2016.04.003

[ref57] Gutiérrez-Sánchez A. Plasencia J. Monribot-Villanueva J. L. Rodríguez-Haas J. B. López-Buenfil J. A. García-Ávila C. J.. (2021). Characterization of the Exo-metabolome of the emergent phytopathogen *Fusarium kuroshium* sp. nov., a causal agent of *Fusarium* dieback. Toxins 13:268. doi: 10.3390/toxins13040268, PMID: 33918546 PMC8069249

[ref58] Haurie V. Perrot M. Mini T. Jenö P. Sagliocco F. Boucherie H. (2001). The transcriptional activator Cat8p provides a major contribution to the reprogramming of carbon metabolism during the diauxic shift in *Saccharomyces cerevisiae*. J. Biol. Chem. 276, 76–85. doi: 10.1074/jbc.M008752200, PMID: 11024040

[ref59] He C. Zhang Z. Li B. Tian S. (2020). The pattern and function of DNA methylation in fungal plant pathogens. Microorganisms 8:227. doi: 10.3390/microorganisms8020227, PMID: 32046339 PMC7074731

[ref60] Hedges D. Proft M. Entian K. D. (1995). CAT8, a new zinc cluster-encoding gene necessary for derepression of gluconeogenic enzymes in the yeast *Saccharomyces cerevisiae*. Mol. Cell. Biol. 15, 1915–1922. doi: 10.1128/MCB.15.4.1915, PMID: 7891685 PMC230417

[ref61] Hirano Y. Kinugasa Y. Asakawa H. Chikashige Y. Obuse C. Haraguchi T.. (2018). Lem2 is retained at the nuclear envelope through its interaction with Bqt4 in fission yeast. Genes Cells 23, 122–135. doi: 10.1111/gtc.12557, PMID: 29292846

[ref62] Hirano Y. Kinugasa Y. Kubota Y. Obuse C. Haraguchi T. Hiraoka Y. (2023a). Inner nuclear membrane proteins Lem2 and Bqt4 interact with different lipid synthesis enzymes in fission yeast. J. Biochem.:mvad017. doi: 10.1093/jb/mvad017, PMID: 36799444

[ref01] Hirano Y. Ohno Y. Kubota Y. Fukagawa T. Kihara A. Haraguchi T.. (2023b). Ceramide synthase homolog Tlc4 maintains nuclear envelope integrity via its Golgi translocation. J. Cell Sci. 136:jcs260923. doi: 10.1242/jcs.260923 37078207

[ref63] Hu C. Inoue H. Sun W. Takeshita Y. Huang Y. Xu Y.. (2019a). Structural insights into chromosome attachment to the nuclear envelope by an inner nuclear membrane protein Bqt4 in fission yeast. Nucleic Acids Res. 47, 1573–1584. doi: 10.1093/nar/gky1186 30462301 PMC6379675

[ref64] Hu C. Inoue H. Sun W. Takeshita Y. Huang Y. Xu Y.. (2019b). The inner nuclear membrane protein Bqt4 in fission yeast contains a DNA-binding domain essential for telomere association with the nuclear envelope. Structure 27, 335–343.e3. doi: 10.1016/j.str.2018.10.010 30503780

[ref65] Huang Y.-T. Skelton J. Hulcr J. (2020). Lipids and small metabolites provisioned by ambrosia fungi to symbiotic beetles are phylogeny-dependent, not convergent. ISME J. 14, 1089–1099. doi: 10.1038/s41396-020-0593-7, PMID: 31988472 PMC7174304

[ref66] Huberman L. B. Wu V. W. Kowbel D. J. Lee J. Daum C. Grigoriev I. V.. (2021). DNA affinity purification sequencing and transcriptional profiling reveal new aspects of nitrogen regulation in a filamentous fungus. Proc. Natl. Acad. Sci. U. S. A. 118:e2009501118. doi: 10.1073/pnas.2009501118, PMID: 33753477 PMC8020665

[ref67] Hulcr J. Barnes I. De Beer Z. W. Duong T. A. Gazis R. Johnson A. J.. (2020). Bark beetle mycobiome: collaboratively defined research priorities on a widespread insect-fungus symbiosis. Symbiosis 81, 101–113. doi: 10.1007/s13199-020-00686-9

[ref68] Ibarra-Juarez L. A. Burton M. A. J. Biedermann P. H. W. Cruz L. Desgarennes D. Ibarra-Laclette E.. (2020). Evidence for succession and putative metabolic roles of Fungi and Bacteria in the farming mutualism of the Ambrosia beetle *Xyleborus affinis*. mSystems 5, e00541–e00520. doi: 10.1128/mSystems.00541-20 32934115 PMC7498683

[ref69] Ibarra-Laclette E. Blaz J. Pérez-Torres C.-A. Villafán E. Lamelas A. Rosas-Saito G.. (2022). Antifungal effect of copper nanoparticles against *Fusarium kuroshium*, an obligate symbiont of *Euwallacea kuroshio* Ambrosia beetle. JoF 8:347. doi: 10.3390/jof8040347, PMID: 35448578 PMC9032953

[ref70] Iyer L. M. Koonin E. V. Aravind L. (2002). Extensive domain shuffling in transcription regulators of DNA viruses and implications for the origin of fungal APSES transcription factors. Genome Biol. 3:1. doi: 10.1186/gb-2002-3-3-research0012 PMC88810 11897024

[ref71] Jamieson K. Rountree M. R. Lewis Z. A. Stajich J. E. Selker E. U. (2013). Regional control of histone H3 lysine 27 methylation in *Neurospora*. Proc. Natl. Acad. Sci. U. S. A. 110, 6027–6032. doi: 10.1073/pnas.1303750110, PMID: 23530226 PMC3625340

[ref72] Jansuriyakul S. Somboon P. Rodboon N. Kurylenko O. Sibirny A. Soontorngun N. (2016). The zinc cluster transcriptional regulator Asg1 transcriptionally coordinates oleate utilization and lipid accumulation in *Saccharomyces cerevisiae*. Appl. Microbiol. Biotechnol. 100, 4549–4560. doi: 10.1007/s00253-016-7356-4, PMID: 26875874

[ref73] John E. Singh K. B. Oliver R. P. Tan K. (2021). Transcription factor control of virulence in phytopathogenic fungi. Mol. Plant Pathol. 22, 858–881. doi: 10.1111/mpp.13056, PMID: 33973705 PMC8232033

[ref74] Jones P. Binns D. Chang H.-Y. Fraser M. Li W. McAnulla C.. (2014). InterProScan 5: genome-scale protein function classification. Bioinformatics 30, 1236–1240. doi: 10.1093/bioinformatics/btu031, PMID: 24451626 PMC3998142

[ref75] Jonkers W. Dong Y. Broz K. Corby Kistler H. (2012). The Wor1-like protein Fgp1 regulates pathogenicity, toxin synthesis and reproduction in the phytopathogenic fungus *Fusarium graminearum*. PLoS Pathog. 8:e1002724. doi: 10.1371/journal.ppat.1002724, PMID: 22693448 PMC3364952

[ref76] Jordá T. Puig S. (2020). Regulation of ergosterol biosynthesis in *Saccharomyces cerevisiae*. Genes 11:795. doi: 10.3390/genes11070795, PMID: 32679672 PMC7397035

[ref77] Jordan P. Choe J.-Y. Boles E. Oreb M. (2016). Hxt13, Hxt15, Hxt16 and Hxt17 from *Saccharomyces cerevisiae* represent a novel type of polyol transporters. Sci. Rep. 6:23502. doi: 10.1038/srep23502 26996892 PMC4800717

[ref78] Joshua I. Höfken T. (2017). From lipid homeostasis to differentiation: old and new functions of the zinc cluster proteins Ecm22, Upc2, Sut1 and Sut2. IJMS 18:772. doi: 10.3390/ijms18040772, PMID: 28379181 PMC5412356

[ref79] Kehelpannala C. Kumar N. S. Jayasinghe L. Araya H. Fujimoto Y. (2018). Naphthoquinone metabolites produced by Monacrosporium ambrosium, the Ectosymbiotic fungus of tea shot-hole borer, *Euwallacea fornicatus*, in stems of tea, *Camellia sinensis*. J. Chem. Ecol. 44, 95–101. doi: 10.1007/s10886-017-0913-1, PMID: 29292470

[ref80] Kinkelin K. Wozniak G. G. Rothbart S. B. Lidschreiber M. Strahl B. D. Cramer P. (2013). Structures of RNA polymerase II complexes with Bye1, a chromatin-binding PHF3/DIDO homologue. Proc. Natl. Acad. Sci. U. S. A. 110, 15277–15282. doi: 10.1073/pnas.1311010110, PMID: 24003114 PMC3780847

[ref81] Kirkendall L. R. Biedermann P. H. W. Jordal B. H. (2015). “Evolution and diversity of bark and Ambrosia beetles” in Bark Beetles. eds. Kirkendall L. R. Biedermann P. H. Jordal B. H. (Amsterdam, Netherlands: Elsevier), 85–156.

[ref82] Kouzminova E. (2001). Dim-2 encodes a DNA methyltransferase responsible for all known cytosine methylation in neurospora. EMBO J. 20, 4309–4323. doi: 10.1093/emboj/20.15.4309, PMID: 11483533 PMC149169

[ref83] Kovalchuk A. Driessen A. J. M. (2010). Phylogenetic analysis of fungal ABC transporters. BMC Genomics 11:177. doi: 10.1186/1471-2164-11-177, PMID: 20233411 PMC2848647

[ref84] Kristensen S. B. Pedersen T. B. Nielsen M. R. Wimmer R. Muff J. Sørensen J. L. (2021). Production and selectivity of key Fusarubins from *Fusarium solani* due to media composition. Toxins (Basel) 13:376. doi: 10.3390/toxins13060376, PMID: 34070644 PMC8230112

[ref85] Langmead B. Salzberg S. L. (2012). Fast gapped-read alignment with bowtie 2. Nat. Methods 9, 357–359. doi: 10.1038/nmeth.1923, PMID: 22388286 PMC3322381

[ref86] Lee K. Hahn J.-S. (2013). Interplay of Aro80 and GATA activators in regulation of genes for catabolism of aromatic amino acids in *Saccharomyces cerevisiae*: regulation of genes for aromatic amino acids catabolism. Mol. Microbiol. 88, 1120–1134. doi: 10.1111/mmi.12246, PMID: 23651256

[ref87] Leslie J. F. Summerell B. A. (2006). The *Fusarium* laboratory manual. Ames, Iowa: Blackwell publ.

[ref88] Letunic I. Bork P. (2018). 20 years of the SMART protein domain annotation resource. Nucleic Acids Res. 46, D493–D496. doi: 10.1093/nar/gkx922 29040681 PMC5753352

[ref89] Li B. Dewey C. N. (2011). RSEM: Accurate transcript quantification from RNA-Seq data with or without a reference genome. Scotts Valley, CA: CreateSpace Independent Publishing Platform. 10.1186/1471-2105-12-323 PMC3163565 21816040

[ref90] Li D. Sirakova T. Rogers L. Ettinger W. F. Kolattukudy P. E. (2002). Regulation of constitutively expressed and induced Cutinase genes by different zinc finger transcription factors in *Fusarium solani* f. sp. pisi (*Nectria haematococca*). J. Biol. Chem. 277, 7905–7912. doi: 10.1074/jbc.M108799200, PMID: 11756444

[ref91] Li M. Yu R. Bai X. Wang H. Zhang H. (2020). *Fusarium*: a treasure trove of bioactive secondary metabolites. Nat. Prod. Rep. 37, 1568–1588. doi: 10.1039/D0NP00038H, PMID: 32785347

[ref92] Liu J. Huang J. Zhao Y. Liu H. Wang D. Yang J.. (2015). Structural basis of DNA recognition by PCG2 reveals a novel DNA binding mode for winged helix-turn-helix domains. Nucleic Acids Res. 43, 1231–1240. doi: 10.1093/nar/gku1351, PMID: 25550425 PMC4333399

[ref93] Livak K. J. Schmittgen T. D. (2001). Analysis of relative gene expression data using real-time quantitative PCR and the 2(-Delta Delta C(T)) method. Methods 25, 402–408. doi: 10.1006/meth.2001.1262, PMID: 11846609

[ref94] López-Díaz C. Rahjoo V. Sulyok M. Ghionna V. Martín-Vicente A. Capilla J.. (2018). Fusaric acid contributes to virulence of *Fusarium* oxysporum on plant and mammalian hosts. Mol. Plant Pathol. 19, 440–453. doi: 10.1111/mpp.12536, PMID: 28093838 PMC6638071

[ref95] Love M. I. Huber W. Anders S. (2014). Moderated estimation of fold change and dispersion for RNA-seq data with DESeq2. Genome Biol. 15:550. doi: 10.1186/s13059-014-0550-8 25516281 PMC4302049

[ref96] Lu S. Wang J. Chitsaz F. Derbyshire M. K. Geer R. C. Gonzales N. R.. (2020). CDD/SPARCLE: the conserved domain database in 2020. Nucleic Acids Res. 48, D265–D268. doi: 10.1093/nar/gkz991, PMID: 31777944 PMC6943070

[ref97] Lynn K. M. T. Wingfield M. J. Durán A. Marincowitz S. Oliveira L. S. S. de Beer Z. W.. (2020). *Euwallacea perbrevis* (Coleoptera: Curculionidae: Scolytinae), a confirmed pest on *Acacia crassicarpa* in Riau, Indonesia, and a new fungal symbiont; *Fusarium rekanum* sp. nov. Antonie Van Leeuwenhoek 113, 803–823. doi: 10.1007/s10482-020-01392-8, PMID: 32086683

[ref98] Malapi-Wight M. Kim J.-E. Shim W.-B. (2014). The N-terminus region of the putative C2H2 transcription factor Ada1 harbors a species-specific activation motif that regulates asexual reproduction in *Fusarium verticillioides*. Fungal Genet. Biol. 62, 25–33. doi: 10.1016/j.fgb.2013.10.008, PMID: 24161731

[ref99] Martín Caballero L. Capella M. Barrales R. R. Dobrev N. Van Emden T. Hirano Y.. (2022). The inner nuclear membrane protein Lem2 coordinates RNA degradation at the nuclear periphery. Nat. Struct. Mol. Biol. 29, 910–921. doi: 10.1038/s41594-022-00831-6, PMID: 36123402 PMC9507967

[ref100] Mayorquin J. S. Carrillo J. D. Twizeyimana M. Peacock B. B. Sugino K. Y. Na F.. (2018). Chemical management of invasive shot hole borer and *Fusarium* dieback in California sycamore (*Platanus racemosa*) in Southern California. Plant Dis. 102, 1307–1315. doi: 10.1094/PDIS-10-17-1569-RE, PMID: 30673581

[ref101] McCulley A. Haarer B. Viggiano S. Karchin J. Feng W. (2014). Chemical suppression of defects in mitotic spindle assembly, redox control, and sterol biosynthesis by hydroxyurea. G3 (Bethesda) 4, 39–48. doi: 10.1534/g3.113.009100 24192836 PMC3887538

[ref102] Medina E. M. Turner J. J. Gordân R. Skotheim J. M. Buchler N. E. (2016). Punctuated evolution and transitional hybrid network in an ancestral cell cycle of fungi. Elife 5:e09492. doi: 10.7554/eLife.09492, PMID: 27162172 PMC4862756

[ref103] Medina E. M. Walsh E. Buchler N. E. (2019). Evolutionary innovation, fungal cell biology, and the lateral gene transfer of a viral KilA-N domain. Curr. Opin. Genet. Dev. 58–59, 103–110. doi: 10.1016/j.gde.2019.08.004 31600629

[ref104] Miller K. E. Inward D. J. G. Gomez-Rodriguez C. Baselga A. Vogler A. P. (2019). Predicting the unpredictable: how host specific is the mycobiota of bark and ambrosia beetles? Fungal Ecol. 42:100854. doi: 10.1016/j.funeco.2019.07.008

[ref105] Morales-Rodríguez C. Sferrazza I. Aleandri M. P. Dalla Valle M. Speranza S. Contarini M.. (2021). The fungal community associated with the ambrosia beetle *Xylosandrus compactus* invading the mediterranean maquis in Central Italy reveals high biodiversity and suggests environmental acquisitions. Fungal Biol. 125, 12–24. doi: 10.1016/j.funbio.2020.09.008, PMID: 33317772

[ref106] Motoyama T. Kadokura K. Ohira T. Ichiishi A. Fujimura M. Yamaguchi I.. (2005). A two-component histidine kinase of the rice blast fungus is involved in osmotic stress response and fungicide action. Fungal Genet. Biol. 42, 200–212. doi: 10.1016/j.fgb.2004.11.002, PMID: 15707841

[ref107] Na F. Carrillo J. D. Mayorquin J. S. Ndinga-Muniania C. Stajich J. E. Stouthamer R.. (2018). Two novel fungal symbionts *Fusarium kuroshium* sp. nov. and *Graphium kuroshium* sp. nov. of Kuroshio shot hole borer (*Euwallacea* sp. nr. *Fornicatus*) cause *Fusarium* dieback on Woody host species in California. Plant Dis. 102, 1154–1164. doi: 10.1094/PDIS-07-17-1042-RE, PMID: 30673440

[ref108] Nødvig C. S. Nielsen J. B. Kogle M. E. Mortensen U. H. (2015). A CRISPR-Cas9 system for genetic engineering of filamentous Fungi. PLoS One 10:e0133085. doi: 10.1371/journal.pone.0133085, PMID: 26177455 PMC4503723

[ref109] Nowrousian M. (2022). The role of chromatin and transcriptional control in the formation of sexual fruiting bodies in Fungi. Microbiol. Mol. Biol. Rev. 86, e00104–e00122. doi: 10.1128/mmbr.00104-22 36409109 PMC9769939

[ref110] O’Donnell K. Sink S. Libeskind-Hadas R. Hulcr J. Kasson M. T. Ploetz R. C.. (2015). Discordant phylogenies suggest repeated host shifts in the *Fusarium*–*Euwallacea ambrosia* beetle mutualism. Fungal Genet. Biol. 82, 277–290. doi: 10.1016/j.fgb.2014.10.014, PMID: 25445310

[ref111] Orikasa Y. Mikumo D. Ohwada T. (2018). A 2-Deoxyglucose-resistant mutant of *Saccharomyces cerevisiae* shows enhanced maltose fermentative ability by the activation of MAL genes. Foods 7:52. doi: 10.3390/foods7040052, PMID: 29614773 PMC5920417

[ref112] Park H.-S. Yu J.-H. (2012). Genetic control of asexual sporulation in filamentous fungi. Curr. Opin. Microbiol. 15, 669–677. doi: 10.1016/j.mib.2012.09.006, PMID: 23092920

[ref113] Patel A. B. Moore C. M. Greber B. J. Luo J. Zukin S. A. Ranish J.. (2019). Architecture of the chromatin remodeler RSC and insights into its nucleosome engagement. elife 8:e54449. doi: 10.7554/eLife.54449, PMID: 31886770 PMC6959994

[ref114] Paysan-Lafosse T. Blum M. Chuguransky S. Grego T. Pinto B. L. Salazar G. A.. (2023). InterPro in 2022. Nucleic Acids Res. 51, D418–D427. doi: 10.1093/nar/gkac993, PMID: 36350672 PMC9825450

[ref115] Pérez-Torres C.-A. Ibarra-Laclette E. Hernández-Domínguez E.-E. Rodríguez-Haas B. Pérez-Lira A.-J. Villafán E.. (2021). Molecular evidence of the avocado defense response to *Fusarium kuroshium* infection: a deep transcriptome analysis using RNA-Seq. PeerJ 9:e11215. doi: 10.7717/peerj.11215, PMID: 33954045 PMC8052963

[ref116] Pinskaya M. Ghavi-Helm Y. Mariotte-Labarre S. Morillon A. Soutourina J. Werner M. (2014). PHD and TFIIS-like domains of the Bye1 transcription factor determine its multivalent genomic distribution. PLoS One 9:e102464. doi: 10.1371/journal.pone.0102464, PMID: 25029256 PMC4100922

[ref117] Pócsi I. Prade R. A. Penninckx M. J. (2004). Glutathione, altruistic metabolite in fungi. Adv. Microb. Physiol. 49, 1–76. doi: 10.1016/S0065-2911(04)49001-8, PMID: 15518828

[ref118] Ramos J. Sychrová H. Kschischo M.. (2016). Yeast membrane transport. Cham: Springer International Publishing.

[ref119] Rassati D. Marini L. Malacrinò A. (2019). Acquisition of fungi from the environment modifies ambrosia beetle mycobiome during invasion. PeerJ 7:e8103. doi: 10.7717/peerj.8103, PMID: 31763076 PMC6870512

[ref120] Raudvere U. Kolberg L. Kuzmin I. Arak T. Adler P. Peterson H.. (2019). G:profiler: a web server for functional enrichment analysis and conversions of gene lists (2019 update). Nucleic Acids Res. 47, W191–W198. doi: 10.1093/nar/gkz369, PMID: 31066453 PMC6602461

[ref121] Reyes A. A. Marcum R. D. He Y. (2021). Structure and function of chromatin Remodelers. J. Mol. Biol. 433:166929. doi: 10.1016/j.jmb.2021.166929, PMID: 33711345 PMC8184634

[ref122] Riquelme M. Aguirre J. Bartnicki-García S. Braus G. H. Feldbrügge M. Fleig U.. (2018). Fungal morphogenesis, from the polarized growth of hyphae to complex reproduction and infection structures. Microbiol. Mol. Biol. Rev. 82, e00068–e00017. doi: 10.1128/MMBR.00068-17 29643171 PMC5968459

[ref123] Rocafort M. Fudal I. Mesarich C. H. (2020). Apoplastic effector proteins of plant-associated fungi and oomycetes. Curr. Opin. Plant Biol. 56, 9–19. doi: 10.1016/j.pbi.2020.02.004, PMID: 32247857

[ref124] Sá-Correia I. Dos Santos S. C. Teixeira M. C. Cabrito T. R. Mira N. P. (2009). Drug:H+ antiporters in chemical stress response in yeast. Trends Microbiol. 17, 22–31. doi: 10.1016/j.tim.2008.09.007, PMID: 19062291

[ref125] Sakai K. Yamaguchi A. Tsutsumi S. Kawai Y. Tsuzuki S. Suzuki H.. (2020). Characterization of FsXEG12A from the cellulose-degrading ectosymbiotic fungus *Fusarium* spp. strain EI cultured by the ambrosia beetle. AMB Expr 10:96. doi: 10.1186/s13568-020-01030-6 PMC7246284 32449090

[ref126] Sánchez-Rangel D. Hernández-Domínguez E.-E. Pérez-Torres C.-A. Ortiz-Castro R. Villafán E. Rodríguez-Haas B.. (2018). Environmental pH modulates transcriptomic responses in the fungus *Fusarium* sp. associated with KSHB *Euwallacea* sp. near *fornicatus*. BMC Genomics 19:721. doi: 10.1186/s12864-018-5083-1 30285612 PMC6167834

[ref127] Sánchez-Rangel D. Sánchez-Nieto S. Plasencia J. (2012). Fumonisin B1, a toxin produced by *Fusarium verticillioides*, modulates maize β-1,3-glucanase activities involved in defense response. Planta 235, 965–978. doi: 10.1007/s00425-011-1555-0, PMID: 22120123

[ref128] Sant D. G. Tupe S. G. Ramana C. V. Deshpande M. V. (2016). Fungal cell membrane-promising drug target for antifungal therapy. J. Appl. Microbiol. 121, 1498–1510. doi: 10.1111/jam.13301, PMID: 27667746

[ref129] Schumacher M. M. Enderlin C. S. Selitrennikoff C. P. (1997). The Osmotic-1 locus of neurospora crassa encodes a putative histidine kinase similar to Osmosensors of Bacteria and yeast. Curr. Microbiol. 34, 340–347. doi: 10.1007/s002849900193, PMID: 9142740

[ref130] Shelest E. (2017). Transcription factors in Fungi: TFome dynamics, three major families, and dual-specificity TFs. Front. Genet. 8:53. doi: 10.3389/fgene.2017.00053 28523015 PMC5415576

[ref131] Short D. P. G. O’Donnell K. Stajich J. E. Hulcr J. Kijimoto T. Berger M. C.. (2017). PCR multiplexes discriminate *Fusarium* symbionts of invasive *Euwallacea* Ambrosia beetles that inflict damage on numerous tree species throughout the United States. Plant Dis. 101, 233–240. doi: 10.1094/PDIS-07-16-1046-RE, PMID: 30682305

[ref132] Six D. L. (2012). Ecological and evolutionary determinants of bark beetle–fungus symbioses. Insects 3, 339–366. doi: 10.3390/insects3010339, PMID: 26467964 PMC4553632

[ref133] Smith R. L. Johnson A. D. (2000). Turning genes off by Ssn6-Tup1: a conserved system of transcriptional repression in eukaryotes. Trends Biochem. Sci. 25, 325–330. doi: 10.1016/S0968-0004(00)01592-9, PMID: 10871883

[ref134] Solovyev V. Kosarev P. Seledsov I. Vorobyev D. (2006). Automatic annotation of eukaryotic genes, pseudogenes and promoters. Genome Biol. 7, S10.1–S10.12. doi: 10.1186/gb-2006-7-s1-s10, PMID: 16925832 PMC1810547

[ref135] Son H. Kim M.-G. Min K. Lim J. Y. Choi G. J. Kim J.-C.. (2014). WetA is required for conidiogenesis and conidium maturation in the ascomycete fungus *Fusarium graminearum*. Eukaryot. Cell 13, 87–98. doi: 10.1128/EC.00220-13, PMID: 24186953 PMC3910957

[ref136] Sperschneider J. Dodds P. N. (2022). EffectorP 3.0: prediction of apoplastic and cytoplasmic effectors in fungi and oomycetes. MPMI 35, 146–156. doi: 10.1094/MPMI-08-21-0201-R, PMID: 34698534

[ref137] Studt L. Wiemann P. Kleigrewe K. Humpf H.-U. Tudzynski B. (2012). Biosynthesis of Fusarubins accounts for pigmentation of *Fusarium fujikuroi* perithecia. Appl. Environ. Microbiol. 78, 4468–4480. doi: 10.1128/AEM.00823-12, PMID: 22492438 PMC3370568

[ref138] Sylvain M.-A. Liang X. B. Hellauer K. Turcotte B. (2011). Yeast zinc cluster proteins Dal81 and Uga3 cooperate by targeting common coactivators for transcriptional activation of Γ-aminobutyrate responsive genes. Genetics 188, 523–534. doi: 10.1534/genetics.110.126003, PMID: 21515579 PMC3176551

[ref139] Takashina K. Chuma I. Kajimura H. Kameyama N. Goto C. Kuroda K. (2020). Pathogenicity and distribution of *Fusarium solani* isolates associated with *Erythrina* decline in Japan. Plant Dis. 104, 731–742. doi: 10.1094/PDIS-01-19-0044-RE, PMID: 31944879

[ref140] Tan K.-C. Oliver R. P. (2017). Regulation of proteinaceous effector expression in phytopathogenic fungi. PLoS Pathog. 13:e1006241. doi: 10.1371/journal.ppat.1006241, PMID: 28426760 PMC5398718

[ref141] Tange Y. Chikashige Y. Takahata S. Kawakami K. Higashi M. Mori C.. (2016). Inner nuclear membrane protein Lem2 augments heterochromatin formation in response to nutritional conditions. Genes Cells 21, 812–832. doi: 10.1111/gtc.12385, PMID: 27334362

[ref142] Teufel F. Almagro Armenteros J. J. Johansen A. R. Gíslason M. H. Pihl S. I. Tsirigos K. D.. (2022). SignalP 6.0 predicts all five types of signal peptides using protein language models. Nat. Biotechnol. 40, 1023–1025. doi: 10.1038/s41587-021-01156-3, PMID: 34980915 PMC9287161

[ref143] Thumuluri V. Almagro Armenteros J. J. Johansen A. R. Nielsen H. Winther O. (2022). DeepLoc 2.0: multi-label subcellular localization prediction using protein language models. Nucleic Acids Res. 50, W228–W234. doi: 10.1093/nar/gkac278, PMID: 35489069 PMC9252801

[ref144] Van der Does H. C. Fokkens L. Yang A. Schmidt S. M. Langereis L. Lukasiewicz J. M.. (2016). Transcription factors encoded on Core and accessory chromosomes of *Fusarium oxysporum* induce expression of effector genes. PLoS Genet. 12:e1006401. doi: 10.1371/journal.pgen.1006401, PMID: 27855160 PMC5140021

[ref145] Verburg K. van Neer J. Duca M. de Cock H. (2022). Novel treatment approach for aspergilloses by targeting germination. J Fungi (Basel) 8:758. doi: 10.3390/jof8080758, PMID: 35893126 PMC9331470

[ref146] Víglaš J. Olejníková P. (2021). An update on ABC transporters of filamentous fungi–from physiological substrates to xenobiotics. Microbiol. Res. 246:126684. doi: 10.1016/j.micres.2020.126684, PMID: 33529790

[ref147] Voigt C. A. Schäfer W. Salomon S. (2005). A secreted lipase of *Fusarium graminearum* is a virulence factor required for infection of cereals: lipase as a virulence factor. Plant J. 42, 364–375. doi: 10.1111/j.1365-313X.2005.02377.x, PMID: 15842622

[ref148] Wagner G. P. Kin K. Lynch V. J. (2012). Measurement of mRNA abundance using RNA-seq data: RPKM measure is inconsistent among samples. Theory Biosci. 131, 281–285. doi: 10.1007/s12064-012-0162-3, PMID: 22872506

[ref149] Wang C. Zhang S. Hou R. Zhao Z. Zheng Q. Xu Q.. (2011). Functional analysis of the Kinome of the wheat scab fungus *Fusarium graminearum*. PLoS Pathog. 7:e1002460. doi: 10.1371/journal.ppat.1002460, PMID: 22216007 PMC3245316

[ref150] Wenderoth M. Pinecker C. Voß B. Fischer R. (2017). Establishment of CRISPR/Cas9 in *Alternaria alternata*. Fungal Genet. Biol. 101, 55–60. doi: 10.1016/j.fgb.2017.03.001, PMID: 28286319

[ref151] Wieczorke R. Krampe S. Weierstall T. Freidel K. Hollenberg C. P. Boles E. (1999). Concurrent knock-out of at least 20 transporter genes is required to block uptake of hexoses in *Saccharomyces cerevisiae*. FEBS Lett. 464, 123–128. doi: 10.1016/S0014-5793(99)01698-1, PMID: 10618490

[ref152] Xie L. Wu Y. Wang Y. Jiang Y. Yang B. Duan X.. (2021). Fumonisin B1 induced aggressiveness and infection mechanism of *Fusarium proliferatum* on banana fruit. Environ. Pollut. 288:117793. doi: 10.1016/j.envpol.2021.117793, PMID: 34274647

[ref153] Xin C. Zhang J. Nian S. Wang G. Wang Z. Song Z.. (2020). Analogous and diverse functions of APSES-type transcription factors in the morphogenesis of the entomopathogenic fungus *Metarhizium rileyi*. Appl. Environ. Microbiol. 86, e02928–e02919. doi: 10.1128/AEM.02928-19 32005738 PMC7117915

[ref154] Xu Y.-J. Singh A. Alter G. M. (2016). Hydroxyurea induces cytokinesis arrest in cells expressing a mutated sterol-14α-demethylase in the ergosterol biosynthesis pathway. Genetics 204, 959–973. doi: 10.1534/genetics.116.191536, PMID: 27585850 PMC5105871

[ref155] Xu D. Xue M. Shen Z. Jia X. Hou X. Lai D.. (2021). Phytotoxic secondary metabolites from Fungi. Toxins (Basel) 13:261. doi: 10.3390/toxins13040261, PMID: 33917534 PMC8067579

[ref156] Yamashiro C. T. Ebbole D. J. Lee B. U. Brown R. E. Bourland C. Madi L.. (1996). Characterization of rco-1 of neurospora crassa, a pleiotropic gene affecting growth and development that encodes a homolog of Tup1 of *Saccharomyces cerevisiae*. Mol. Cell. Biol. 16, 6218–6228. doi: 10.1128/MCB.16.11.6218, PMID: 8887652 PMC231625

[ref157] Yang Y. Huang P. Ma Y. Jiang R. Jiang C. Wang G. (2022). Insights into intracellular signaling network in *Fusarium* species. Int. J. Biol. Macromol. 222, 1007–1014. doi: 10.1016/j.ijbiomac.2022.09.211, PMID: 36179869

[ref158] Yu J.-H. Hamari Z. Han K.-H. Seo J.-A. Reyes-Domínguez Y. Scazzocchio C. (2004). Double-joint PCR: a PCR-based molecular tool for gene manipulations in filamentous fungi. Fungal Genet. Biol. 41, 973–981. doi: 10.1016/j.fgb.2004.08.001, PMID: 15465386

[ref159] Yu H. Yang H. Haridas S. Hayes R. D. Lynch H. Andersen S.. (2023). Conservation and expansion of transcriptional factor repertoire in the *Fusarium oxysporum* species complex. J Fungi (Basel) 9:359. doi: 10.3390/jof9030359, PMID: 36983527 PMC10056406

[ref160] Yuan G. F. Fu Y. H. Marzluf G. A. (1991). Nit-4, a pathway-specific regulatory gene of neurospora crassa, encodes a protein with a putative binuclear zinc DNA-binding domain. Mol. Cell Biol. 11, 5735–5745. doi: 10.1128/mcb.11.11.5735-5745.1991 1840634 PMC361945

[ref161] Zeng Y. Song H. Xia L. Yang L. Zhang S. (2023). The responses of poplars to fungal pathogens: a review of the defensive pathway. Front. Plant Sci. 14:1107583. doi: 10.3389/fpls.2023.1107583, PMID: 36875570 PMC9978395

[ref162] Zhang S. Zhu P. Cao B. Ma S. Li R. Wang X.. (2021). An APSES transcription factor Xbp1 is required for Sclerotial development, appressoria formation, and pathogenicity in *Ciboria shiraiana*. Front. Microbiol. 12:739686. doi: 10.3389/fmicb.2021.739686, PMID: 34646256 PMC8503677

[ref163] Zhao S. An B. Guo Y. Hou X. Luo H. He C.. (2020). Label free proteomics and systematic analysis of secretome reveals effector candidates regulated by SGE1 and FTF1 in the plant pathogen *Fusarium oxysporum* f. sp. cubense tropical race 4. BMC Genomics 21:275. doi: 10.1186/s12864-020-6695-9 32245409 PMC7119298

[ref164] Zhao Y. Su H. Zhou J. Feng H. Zhang K.-Q. Yang J. (2015). The APSES family proteins in fungi: characterizations, evolution and functions. Fungal Genet. Biol. 81, 271–280. doi: 10.1016/j.fgb.2014.12.003, PMID: 25534868

[ref165] Zuriegat Q. Zheng Y. Liu H. Wang Z. Yun Y. (2021). Current progress on pathogenicity-related transcription factors in *Fusarium oxysporum*. Mol. Plant Pathol. 22, 882–895. doi: 10.1111/mpp.13068, PMID: 33969616 PMC8232035

