# Supplementary material for: The biological relevance of the FspTF transcription factor, homologous of Bqt4, in Fusarium sp. associated with the ambrosia beetle Xylosandrus morigerus
Source: Front Microbiol. 2023 Jul 14;14:1224096. doi: 10.3389/fmicb.2023.1224096 (PMC10375492; doi:10.3389/fmicb.2023.1224096)
Supplement: Supplementary file 12 [file Data_Sheet_1.PDF]

Supplementary Material

The biological relevance of the FspTF transcription factor, homologous of Bqt4, in *Fusarium* sp. associated with the ambrosia beetle *Xylosandrus morigerus.*

Nohemí Carreras-Villaseñor, Luis A. Martínez-Rodríguez, Enrique Ibarra-Laclette, Juan L. Monribot-Villanueva, José B. Rodríguez-Haas, José A. Guerrero-Analco and Diana Sánchez-Rangel*

*** Correspondence:** Diana Sánchez-Rangel, diana.sanchez@inecol.mx

1 Supplementary Figures and Tables

1.1 Supplementary Figures

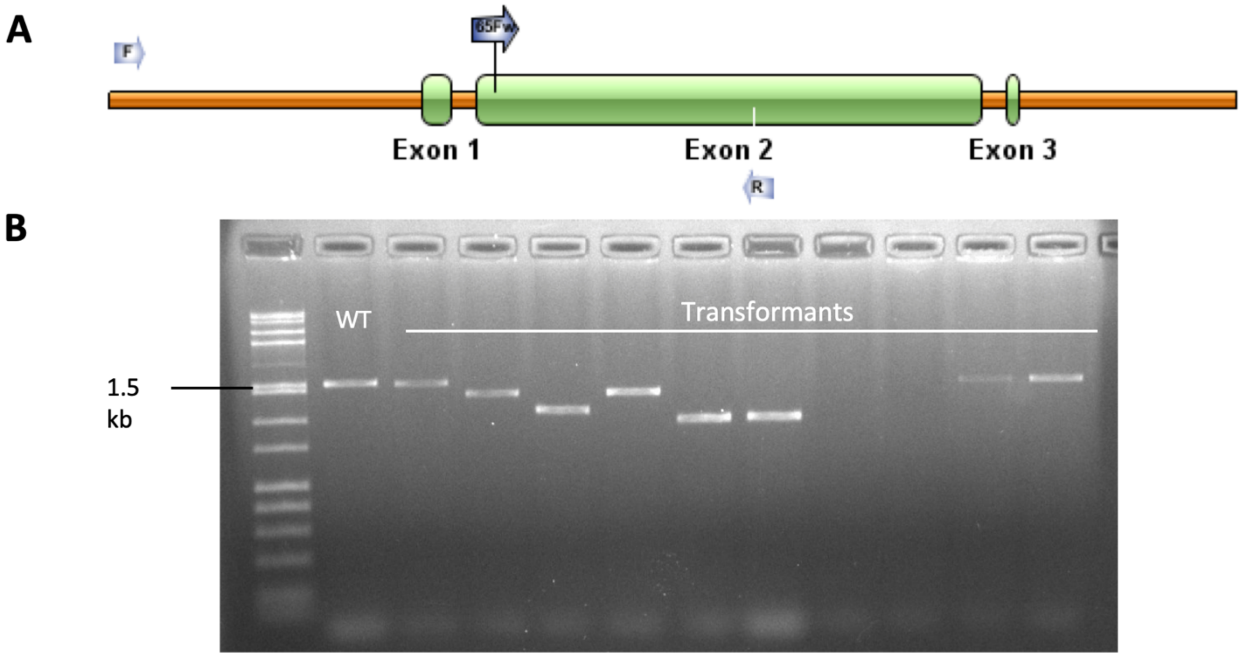

Supplementary Figure S1. Gene Edition analysis of *tf* by PCR in different transformant strains. (A) Annealing sites of primers for gene edition analysis. FT_CRISPR-1_F primer (F) anneals 979 bp up-stream and FT_CRISPR-1_R primer (R) anneals 573 pb down-stream of the protospacer sequence (arrow 65Fw). **(B)** Electroforesis of PCR products of WT and transformants strains showing different sized fragments.

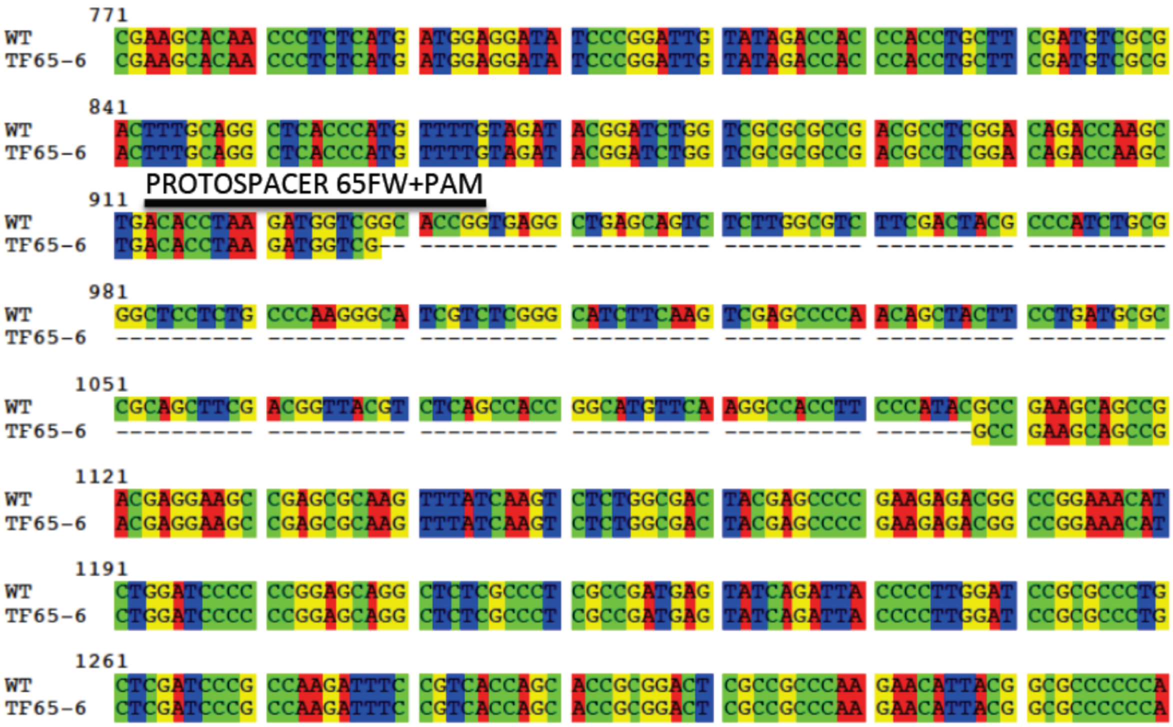

Supplementary Figure S2. Sequence alignment analysis of *Fusarium* sp. INECOL_BM-06 WT and TF65-6 strains revealed a 179 bp deletion (dotted line). The sequence of the protospacer+PAM is indicated.

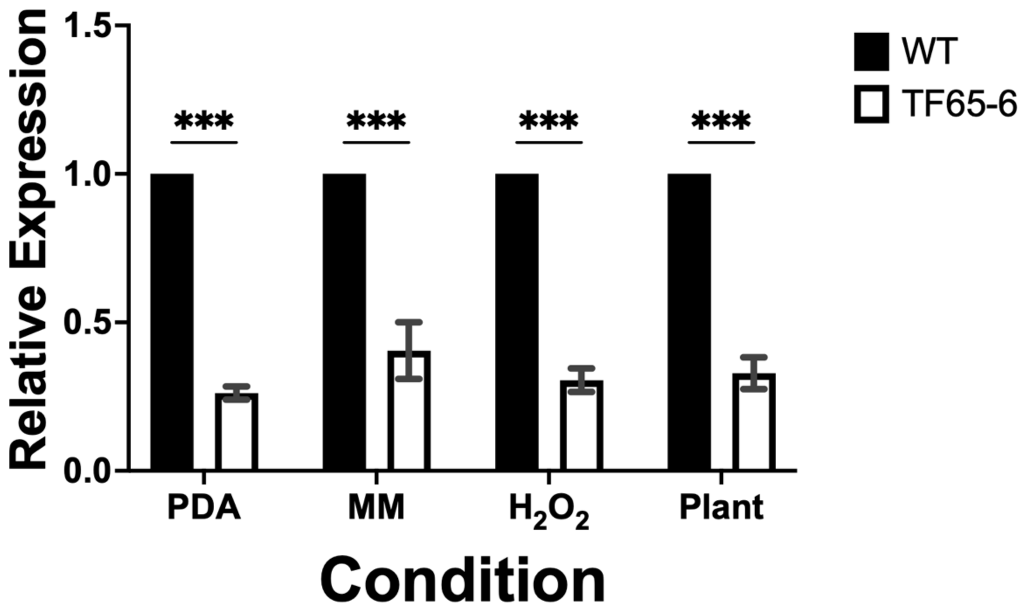

Supplementary Figure S3. Expression of *tf* in *Fusarium* sp. INECOL_BM-06 WT and TF65-6. Expression of Fsp*tf* in WT and TF65-6 grown in different conditions. Expression is relative to *EIF1-a*

expression. Expression in WT was set in 1. Bars are average ± SD of 3 technical replicates. (***p-value<0.001 by Two-way ANOVA with Bonferroni multiple comparison correction).

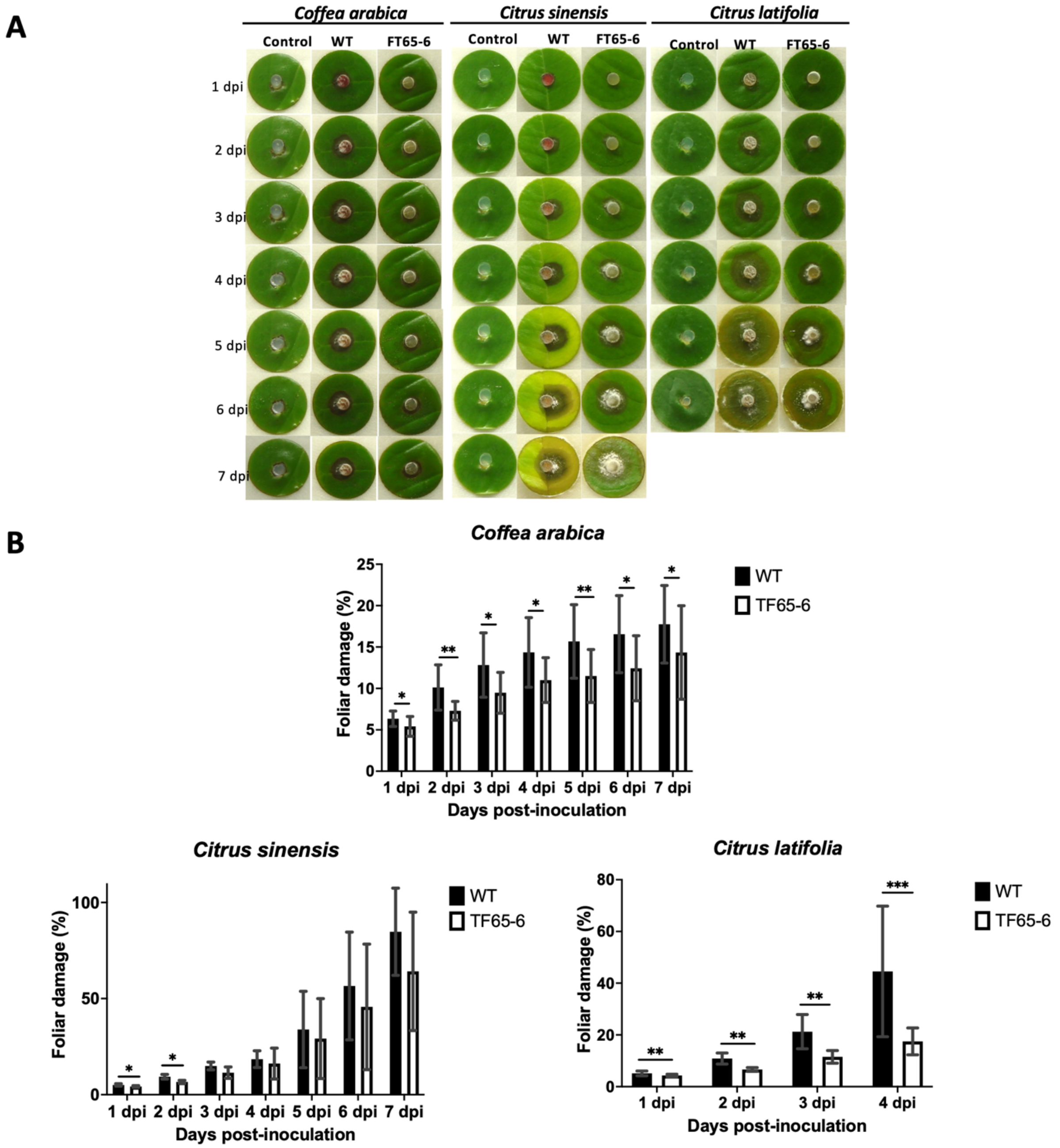

Supplementary Figure S4. FspTF regulates pathogenesis in *Fusarium* sp. INECOL_BM-06. (A) Infection progression during 7 dpi in foliar disc of *Coffea arabica, Citrus sinensis* and, during 6 dpi in foliar disc of *Citrus latifolia* inoculated with *Fusarium* sp. INECOL_BM-06 WT and TF65-6. **(B)** Progression of the percentage of damage during 7 dpi of the foliar disc of *Coffea arabica, Citrus sinensis* and, during 4 dpi of foliar disc of *Citrus latifolia* inoculated with *Fusarium* sp. INECOL_BM-06 WT and TF65-6. Bars are average ± SD of at least 10 technical replicates. (***p-value<0.001, **p-value<0.01, *p-value<0.05 by Two-way ANOVA with Bonferroni multiple comparison correction).

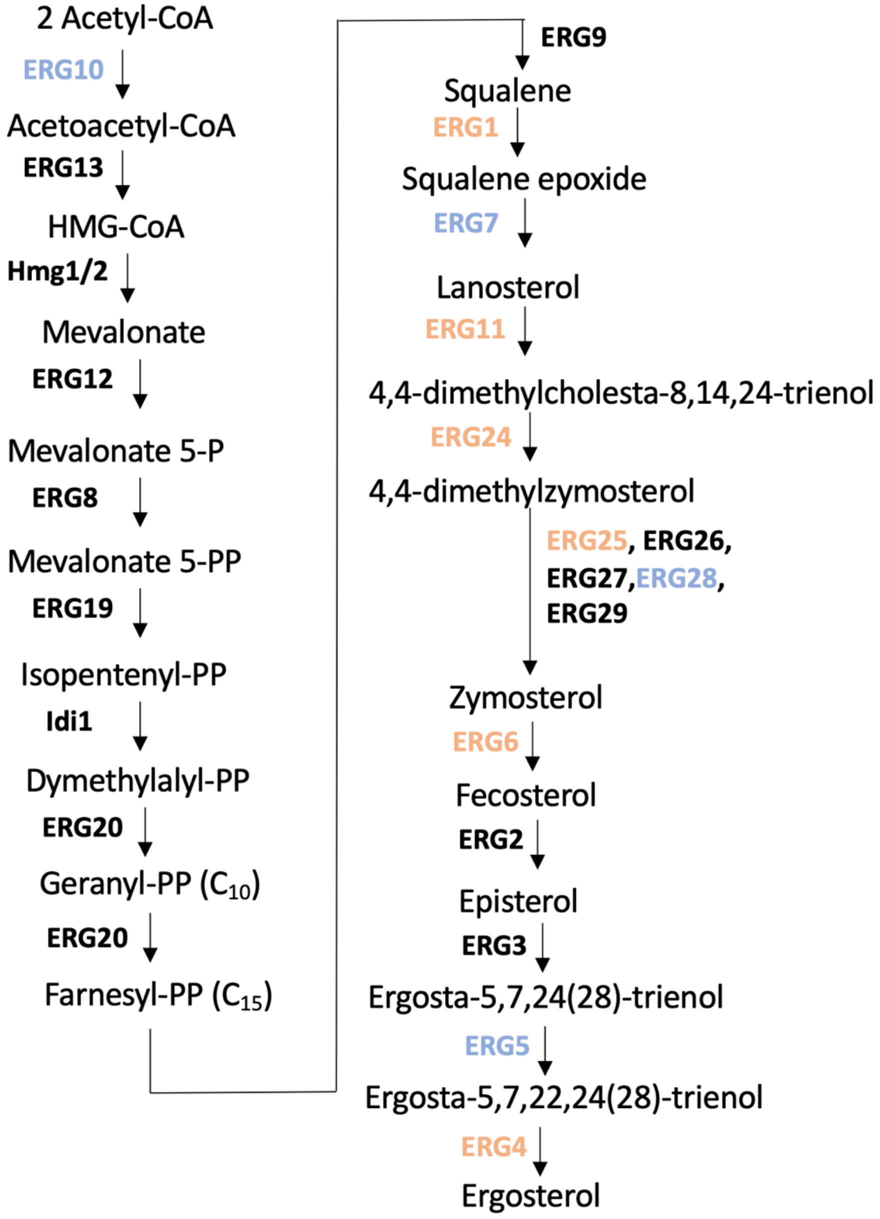

Supplementary Figure S5. FspTF regulates ergosterol biosynthetic pathway *Fusarium* sp. INECOL_BM-06. The different color in enzymes names indicates expression profile in *Fusarium* sp. TF65-6. Blue: Repressed. **Black**: No differential. Orange: Induced.

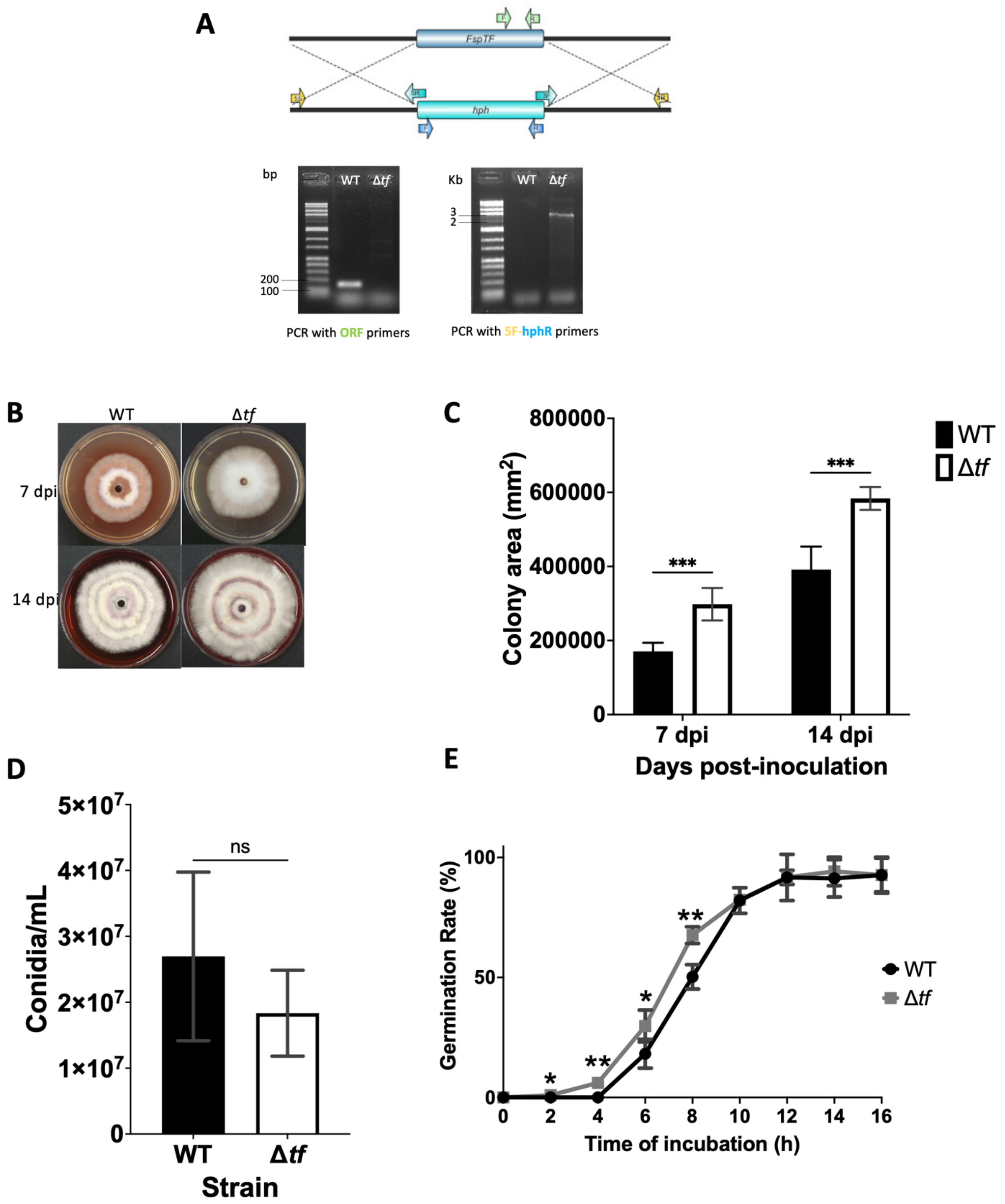

Supplementary Figure S6. FspTF regulates development in *Fusarium* sp. INECOL_BM-06. (A) Diagram of gene replacement construction and genomic DNA PCR verification of *Fusarium* sp. INECOL_BM-06 Δ*tf*. **(B)** Growth of *Fusarium* sp. INECOL_BM-06 WT and Δ*tf* in PDA. **(C)** Colony area of *Fusarium* sp. INECOL_BM-06 WT and Δ*tf* in PDA. Data obtained at 7 and 14 dpi. Bars are average ± SD of 2 biological replicates with 4 technical replicates each one. **(D)** Conidia quantification in *Fusarium* sp. INECOL_BM-06 WT and Δ*tf*. Conidia were obtained after 7 days of incubation in liquid CMC. Bars are average ± SD of 3 biological replicates with 3 technical replicates each one. **(E)**

Germination rates of *Fusarium* sp. INECOL_BM-06 WT and Δ*tf* when inoculated in liquid MM at 28°C. The number of conidia showing germ-tube protrusion was recorded at 2 h intervals and which is represented as a percentage of the total number of conidia counted with the hemocytometer. Data are presented as the average ± SD of three independent experiments with two technical replicates each one. (***p-value<0.001, **p-value<0.01, *p-value<0.05 by Two-way ANOVA with Bonferroni multiple comparison correction).

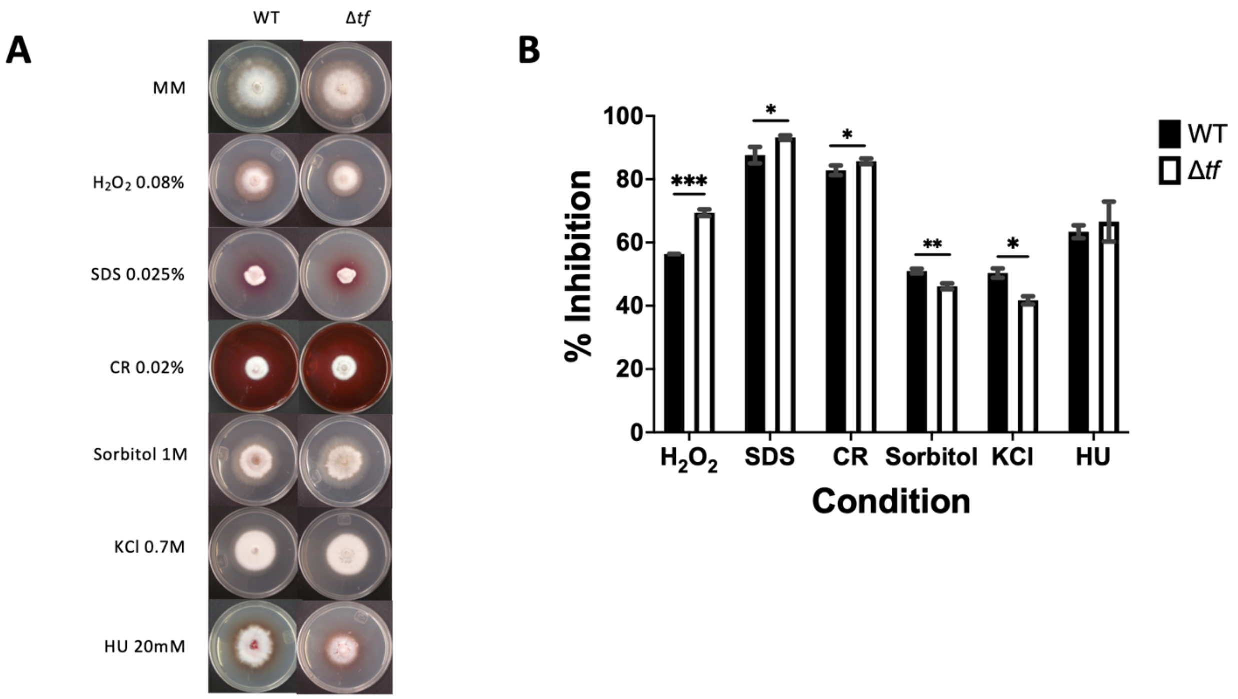

Supplementary Figure S7. Response to different stress conditions of *Fusarium* sp. INECOL_BM-06 and Δ*tf*. (A) Growth of *Fusarium* sp. INECOL_BM-06 WT and Δ*tf* in MM amended with H_2O_2 0.08%, Sorbitol 1M, SDS 0.025%, CR 0.02%, KCl 0.7M or HU 20mM. **(B)** Mycelial growth inhibition of INECOL_BM-06 WT and Δ*tf* grown in MM amended with H_2O_2 0.08%, Sorbitol 1M, SDS 0.025%, CR 0.02%, KCl 0.7M or HU 20mM. Data obtained at 7 dpi. Bars are average ± SD of three technical replicates. (***p-value<0.001, **p-value<0.01, *p-value<0.05 by Two-way ANOVA with Bonferroni multiple comparison correction).

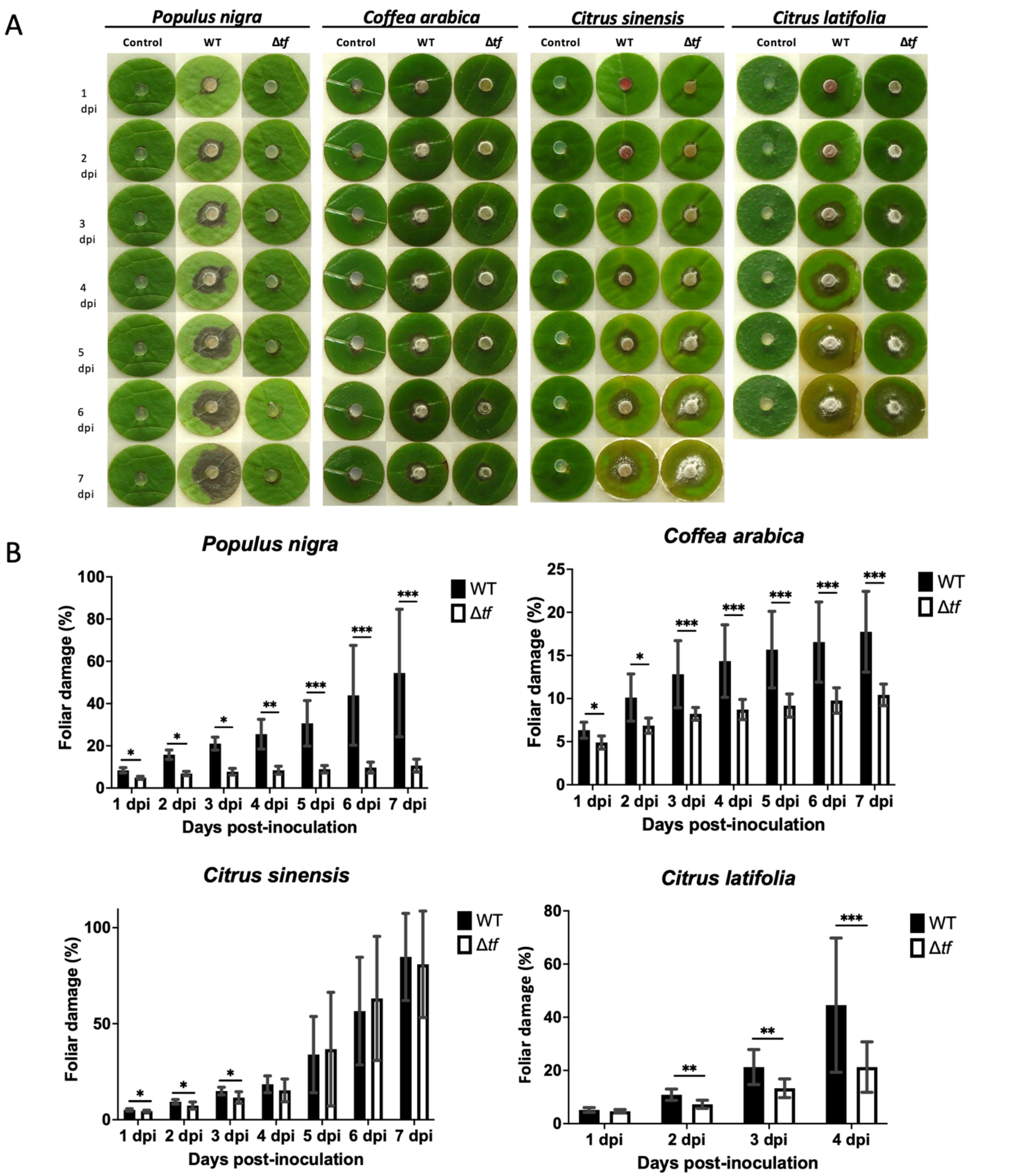

Supplementary Figure S8. FspTF regulates pathogenesis in *Fusarium* sp. INECOL_BM-06. (A) Infection progression during 7 dpi in foliar disc of *Populus nigra, Coffea arabica, Citrus sinensis* and, during 6 dpi in foliar disc of *Citrus latifolia* inoculated with *Fusarium* sp. INECOL_BM-06 WT and Δ*tf*. **(B)** Progression of the percentage of damage during 7 dpi of the foliar disc of *Populus nigra, Coffea arabica, Citrus sinensis* and, during 4 dpi of foliar disc of *Citrus latifolia* inoculated with *Fusarium* sp. INECOL_BM-06 WT and Δ*tf*. Bars are average ± SD of at least 10 technical replicates. (***p-

value<0.001, **p-value<0.01, *p-value<0.05 by Two-way ANOVA with Bonferroni multiple comparison correction).
